# Recent Advances in Egg White Biotechnology, Microbiology, and Quality

**DOI:** 10.1111/1541-4337.70495

**Published:** 2026-05-18

**Authors:** Insa Mannott, Ramona Bosse, Monika Gibis

**Affiliations:** ^1^ Department of Food Technology, Institute EcoMaterials Bremerhaven University of Applied Sciences Bremerhaven Germany; ^2^ Department of Food Material Science, Institute of Food Science and Biotechnology University of Hohenheim Stuttgart Germany

**Keywords:** egg white peptides, egg white proteins, egg white, fermentation, food safety, lactic acid bacteria, pathogens, processing, spoilage, thermal treatment

## Abstract

International trade in egg products has increased in recent decades to meet the rising demands of a global population, the food industry, and consumers. Egg white is a rich source of proteins and peptides suitable for various food applications. Processing egg whites is important in the food industry. This review thoroughly examines different aspects of egg white technology, including the definition of egg white, the natural microflora of eggs, and potential contaminants that may be present during processing. The impact of raw materials on the quality and features of egg products is also discussed, along with traditional and modern processing technologies regarding their applicability and efficiency. The study shows recent advancements and innovative approaches that could enhance egg white processing. Additionally, the fermentation of egg whites is explored as a promising method to modify their functional properties and produce bioactive compounds. The review shows the health benefits and significance of bioactive peptides derived from egg white in the development of functional foods. Overall, this review provides a comprehensive overview of current research in egg white processing, demonstrating how this valuable raw material can be utilized to create innovative products with enhanced nutritional and functional properties.

**Abbreviations**
CP: cold plasma
EFSA: European Food Safety Authority
EU: European Union
FAO: Food and Agriculture Organization
HHP: high hydrostatic pressure
LAB: lactic acid bacteria
MW: microwave
PEF: pulsed electric fields
TMA: trimethylamine
USA: United States of America
USDA: United States Department of Agriculture
UV: ultraviolet light
WHO: World Health Organization

## 1 Introduction

Poultry and its products (meat and eggs) have been a major source of protein for human diets. Chicken eggs can be eaten worldwide without religious restrictions and are an affordable, highly bioavailable source of proteins and nutrients (Miranda et al. 2015).

Several essential amino acids of high biological value (e.g., histidine and tryptophan) are absorbed through egg consumption (Stefanova et al. 2021; Puglisi and Fernandez 2022).

Furthermore, eggs are an ingredient in many food products. Due to their functional properties, especially their coagulating, thickening, foaming, and emulsifying abilities, eggs are used in various foods like cakes, meringues, and mayonnaise (Lesnierowski and Stangierski 2018; Stefanova et al. 2021).

Global egg production rose from 61.7 million tons in 2008 to 86.7 million tons in 2020. Asia was the leading producer of shell eggs in 2020, accounting for 62.4%, followed by Europe (12.8%), Latin America (12%), and the United States and Canada (8.3%) (FAO 2024; Molnár and Szőllősi 2020). The shift toward flexitarian diets, increased use of meat substitutes alongside vegetarian diets, and the popularity of “low carb” diets have driven a rise in egg consumption. On average, 20% of the total egg intake in Europe consists of egg products (Gautron et al. 2022). According to the United States Department of Agriculture (USDA), approximately 93.7 billion eggs were consumed in the United States in 2022, with 29.7% of these being egg products (USDA 2023).

As high meat consumption is considered problematic from the perspective of a sustainable and healthy diet, vegetarianism is becoming increasingly popular (Parodi et al. 2018; Saari et al. 2021). Eggs are more sustainable than cattle and dairy; they cause fewer greenhouse gas emissions. As egg production uses less water and land than cattle and dairy farming, egg whites are a promising source of sustainable protein (Poore and Nemecek 2018). These ecological advantages make egg whites a promising source of protein for developing sustainable food products.

Due to health concerns and demand, egg consumption has risen. Consequently, there is a growing interest in researching ways to improve the efficiency and sustainability of egg‐derived ingredients, especially egg whites. However, much work remains to be done on the sustainability of egg products (Molnár and Szőllősi 2020).

Egg white is a highly used product that is comparable to yolk in terms of value and versatility (Li et al. 2021; Razi et al. 2023). Due to its high protein, low fat, and low carbohydrate content, egg white is ideal for producing health‐oriented foods (Jäger et al. 2017; Razi et al. 2023; Miranda et al. 2015).

To strengthen the market position of egg manufacturers, research into functional egg ingredients and the development of new products are required (Lesnierowski and Stangierski 2018).

Despite numerous individual studies on microbiological hazards in specific types of egg product, an overview of the impact of processing steps on the functional properties of egg white and the associated biotechnological risks is lacking. This review aims to compile existing scientific research on the microbial safety, functional properties, quality, and biotechnology of processed egg white products.

## 2 Methodology

This review follows the PRISMA‐ScR (Preferred Reporting Items for Systematic Reviews and Meta‐Analyses extension for Scoping Reviews) guideline (Page et al. 2021). The protocol is based on the checklist by Tricco et al. (2018). The methodology and relevant protocols are included in Sections S1–S3.

## 3 Definition and Characterization of Egg White

### 3.1 Definition of Egg Products

According to European and United States regulations, egg products can only be used to produce food for human consumption. Eggs that meet the criteria for commercial Grade A being free of cracks or blood inclusions and clean, intact, and properly colored can be sold as shell eggs. Eggs weighing less than 53 g may not be sold as shell eggs. Those that do not meet these criteria are processed into egg products (European Union 2004; USDA 2023).

Products derived from eggs are defined as processed products obtained through the processing of eggs or various components, mixtures of eggs, or subsequent processing of such processed products (European Union 2004; Mattisek and Fischer 2021; USDA 2023).

**FIGURE 1:**
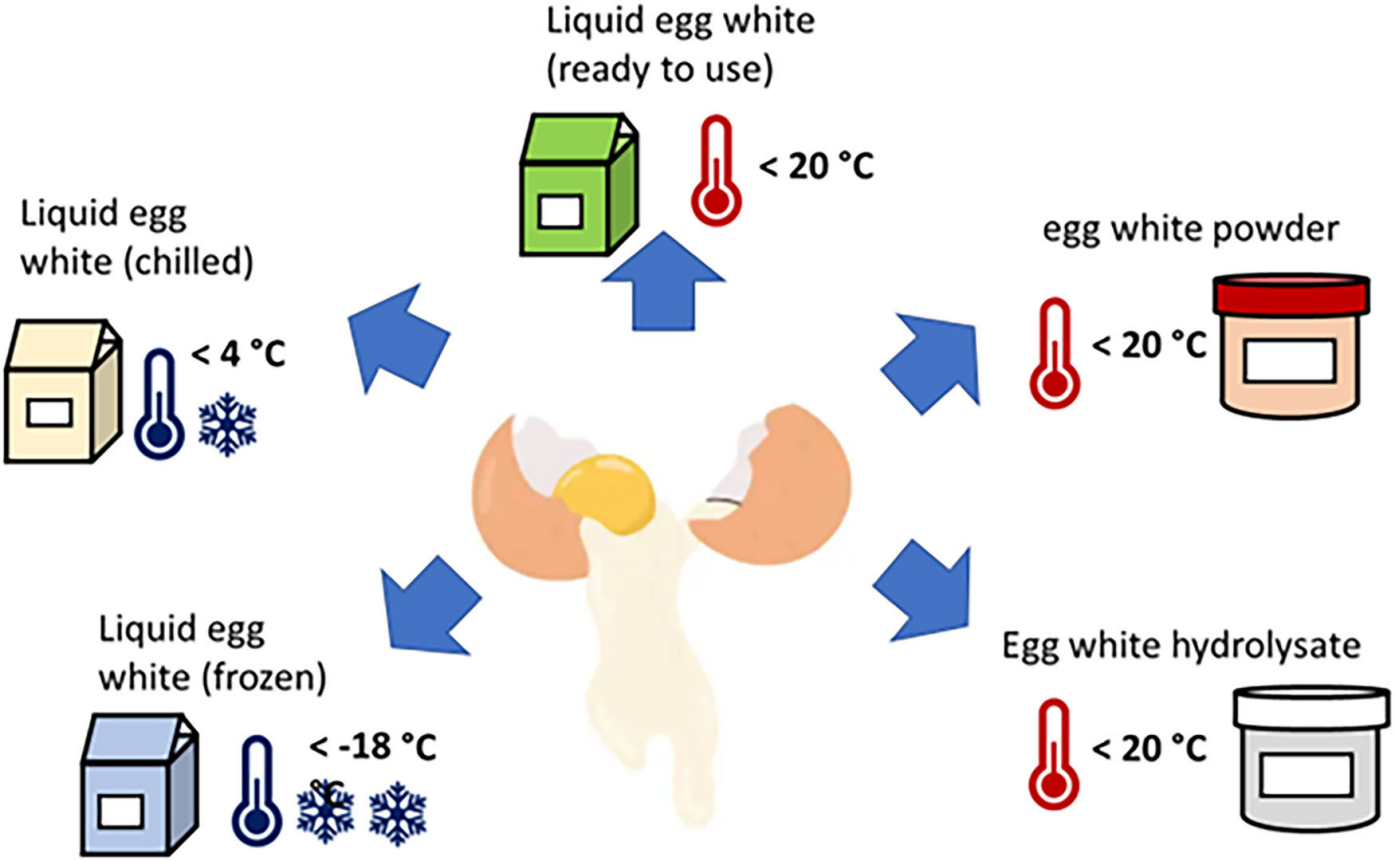
Products from egg white and their storage temperatures (own representation, based on Katekhong and Charoenrein 2017, 2018; Wei et al. 2020; Zhang, Zhu et al. 2023; Zhang, Tang et al., 2023; Zang et al. 2024; Gautron et al. 2021; Razi et al. 2023; Lesnierowski and Stangierski 2018).

Egg products are obtained as whole egg, egg white, or egg yolk and are further processed into liquid, frozen, chilled, ready‐to‐use, or dried products (Figure 1). These are used in manufacturing bakery products, confections, dry soups, and ice cream. Egg products are from hens’ eggs only (Lesnierowski and Stangierski 2018; Razi et al. 2023).

The egg white makes up 56% of the total protein content of eggs. It is mainly water (about 88%), with approximately 10% of the proteins, carbohydrates (∼1%), minerals, and vitamins, such as niacin, riboflavin, magnesium, potassium, and sodium (Gautron et al. 2021; Razi et al. 2023; Liao et al. 2018; Stefanova et al. 2021). Egg whites contain minimal fat and cholesterol, making them a suitable ingredient for the food industry. This ingredient is known for its high quality, nutritional richness, and bioavailability, thanks to the high biological value of the protein (Lesnierowski and Stangierski 2018; Liao et al. 2018). As a result, egg protein is assigned a reference biological value of 100. Only consuming a combination of different foods has been shown to produce a higher biological value (Kumar et al. 2017; Matsuoka et al. 2019). Therefore, egg white plays an important role in the global human diet.

### 3.2 Egg White Products

Alongside the nutritional relevance of eggs, their use has increasingly expanded beyond shell eggs toward processed egg products, particularly in the food industry (Baba et al. 2018). When processing egg products, ensuring microbiological safety is paramount, with consistent quality, product safety, and rapid processing being among the most critical requirements (Gautron et al. 2021). Because of the functional properties of eggs, food companies have increased their demand for suitable egg‐based protein sources such as egg white to incorporate into their products, such as vegetarian sausage, due to the texture‐stabilizing properties (Razi et al. 2023).

Liquid egg whites are available chilled, ready‐to‐use, or frozen (Figure 1). Frozen egg whites have a longer shelf life, and manufacturers can thaw and use them as needed. Dried egg whites are more convenient than fresh or liquid egg whites because they have a longer shelf life (up to 549 days at room temperature) and are stable during storage (Katekhong and Charoenrein 2017, 2018; Wei et al. 2020; Zhang, Tang et al. 2023; Zang et al. 2024). Additionally, egg white hydrolysates are gaining popularity because of their enhanced functional properties (Loveday 2023; Nasri 2017).

### 3.3 Microbiological Susceptibility of Egg Products

Egg products must undergo processing and pasteurization because of their microbiological susceptibility. The risk of microbial contamination varies between types of egg products due to differences in processing, storage conditions, initial contamination, and physical properties. Liquid egg products are susceptible to microbial contamination due to their high moisture content (*a*_W_‐value 0.95, water activity) and pH level. These factors create an environment that allows the growth of microorganisms (Guillén et al. 2024; Kang et al. 2021).

Pathogenic microorganisms can survive and grow in egg products if proper handling procedures are not followed (Ricke et al. 2023). Pasteurization is a step in ensuring the safety of liquid egg products by eliminating these pathogens (Dong et al. 2024). Pasteurized liquid egg whites should be kept at temperatures of ≤4°C, with a shelf life of a few weeks to 1 month (European Union 2004; Techer et al. 2014).

Frozen egg products pose a lower risk of microbial contamination due to lower temperatures (<−18°C), which inhibit the growth of most microorganisms (Zhang et al. 2025). However, the risk of microbial contamination remains, especially with improper handling, such as repeated thawing and freezing, which provides an opportunity for surviving bacteria to recover (Harrison et al. 2013; Ehuwa et al. 2021). Furthermore, psychrotrophic microorganisms, such as *Listeria monocytogenes* (*L. monocytogenes*), can withstand low temperatures and become active during improper storage (Fay et al. 2024). The low water activity (*a*_W_‐value <0.3) of dried egg products reduces the risk of microbial contamination. However, moisture can enable microorganisms to proliferate in dried egg products, potentially compromising their safety and quality. *Bacillus cereus* (*B. cereus*) poses a particular risk because its spores can survive the drying process and grow in moist environments (Cho and Chung 2020). Inadequate storage conditions and increased humidity can lead to mold growth. Some molds, like *Aspergillus* spp. and *Penicillium* spp., can pose health risks (Snyder and Worobo 2018; Avery et al. 2019). However, if egg white powder is stored in a dry, cool place, protected from light, it can be stored 1 year or longer.

Microbial contamination is highest in liquid egg white, followed by frozen and dried egg white products, but necessary preservation methods have an influence on protein functionality, as discussed below.

## 4 Influence of Microorganisms on Eggs and Egg White

### 4.1 Intrinsic Factors

#### 4.1.1 Autochthonous Microflora of the Eggshell

Eggs are usually free of microorganisms in freshly laid eggs from healthy hens. The egg contains a series of natural antimicrobial substances that protect it from contamination for a certain period. These protective effects are concentration‐dependent and can diminish over time or under certain conditions, which increases the risk of microbial contamination (Jabalera et al. 2022).

The eggshell, on the other hand, is colonized by microorganisms immediately after laying. The microbiota on the eggshell is usually similar to that of the environment (Table 1). However, some of the native microorganisms can act as a protective film, and the extent of colonization depends on the environmental type (Aygun 2017; Grizard et al. 2014). Despite this potential protective effect, several studies have shown that bacteria can penetrate the eggshell and its membranes, thereby contaminating the egg contents. During laying, eggshells can be colonized with both pathogenic and non‐pathogenic microorganisms. The microbial activity on the eggshell depends on various factors such as farming methods, the breed and age of hens, and the season (Aygun 2017; Chen, Li, et al. 2019; Vlčková et al. 2019). Studies show that eggshells harbor a complex microbial community that includes bacteria such as *Pseudomonas* spp., *Alcaligenes* spp., *Proteus* spp., *Arthrobacter* spp., *Serratia* spp., *Aeromonas* spp., *Hafnia* spp., *Citrobacter* spp., *Salmonella* spp., *Micrococcus* spp., *Staphylococcus* spp., *Bacillus* spp., and *Lactobacillus* spp. (Aygun 2017). Additionally, fungi such as *Penicillium* spp., *Aspergillus* spp., *Rhizopus* spp., *Mucor* spp., and *Cladosporium* spp., along with the yeast *Rhodotorula* spp., have been detected on eggshells (Tomczyk et al. 2018).

**TABLE 1:** Characteristics of the main antimicrobial proteins of chicken egg white (ni = not indicated; (Gram(+) = Gram‐positive; Gram(−) = Gram‐negative; Vit. = vitamin).

Egg white protein	Fraction of the protein from fresh egg white [%]	Denaturation temperature [°C]	Predicted or confirmed antimicrobial activity	Inhibited microorganism species	Log reduction	References
Avidin	0.05	70–85	Chelation of biotin makes it inaccessible for bacteria; growth inhibition	various Gram(−) and Gram(+) bacteria, *Escherichia coli, Klebsiella pneumoniae, Serratia marcescens, Pseudomonas aeruginosa, Staphylococcus aureus*, and *Staphylococcus epidermidis*	ni	Julien et al. (2019), Moreau et al. (2022)
Cystatin	0.05	ni	Cysteine endopeptidase inhibitor (such as papain and ficin) Antioxidant properties	Gram(+) and Gram(−) bacteria for example *S. aureus, Bacillus subtilis, Porphyromonas gingivalis, Candida* spp., *Acinetobacter iwofii*, *E. coli*, and *P. aeruginosa*	ni	Kupaj et al. (2020), Anton et al. (2016), Liao et al. (2018)
Lysozyme	3.5	69–77	Hydrolysis of β(1–4)‐glycosidic bonds in bacterial cell wall peptidoglycan acts specifically on the polymer *n*‐acetyl glucosamine *n*‐acetylmuramic acid, cleaving the link between them Flocculation of bacterial cells Formation of oligosaccharides from bacterial cell walls, tetrasaccharides by transglycosylation Antioxidant properties	Gram(+) bacteria	2 mg/mL lysozyme 1.6 log (*E. coli*) at pH 6, no reduction at pH 7 8 log (*Staphylococcus carnosus*) at pH 6 and 7	Liao et al. (2018), Vilcacundo et al. (2018), Stefanova et al. (2021)
Ovalbumin	54	75–84	Binds metal ions with SH‐groups Antioxidant properties	ni	5–50 mg/mL ovoalbumin, 11%–60% (*E. coli*), 20%–55% (*S. aureus*)	Nimalaratne and Wu (2015), Stefanova et al. (2021), Julien et al. (2019), Arzumanyan et al. (2019)
Ovoflavo‐protein	0.8	ni	Chelation of riboflavin (or Vit. G or Vit. B2); this makes it inaccessible to bacteria that need it Serine protease inhibitor	ni	ni	Anton et al. (2016), Mine (2014)
Ovoinhibitor		ni	Inhibition of various proteases (including trypsin, chymotrypsin, and fungal proteinase) Antioxidant properties	*Bacillus thuringiensis*	ni	Yao et al. (2020), Liao et al. (2018)
Ovomucoid	13	77	Inhibition of trypsin Protease inhibitor	ni	Biofilm reduction of *Listeria monocytogenes* and *S. aureus*	Liao et al. (2018), Stefanova et al. (2021)
Ovotransferrin (conalbumin)	12	61–65	Chelating metal ions (especially Fe^3+^, but also Cu^3+^, Mn^2+^, Co^2+^, Cd^2+^, Zn^2+^, and Ni^2+^) Antioxidant properties	Gram(+) more sensitive than Gram(−), *S. aureus*, *Bacillus cereus*, *L. monocytogenes*, *Helicobacter pylori*, *Pseudomonas* spp., *E. coli*, *Streptococcus mutans*, *Candida* spp.	6.1 log (*B. cereus*) at pH 9.2	Julien et al. (2019), Rathnapala et al. (2021), Stefanova et al. (2021)

Studies using advanced sequencing techniques (16S rRNA) have identified a range of microorganisms associated with eggs, including bacteria, fungi, and viruses. The main bacterial groups are *Firmicutes* spp., *Proteobacteria* spp., and *Actinobacteria* spp., whereas fungi, like *Penicillium* spp. and *Aspergillus* spp., are frequently detected (Techer et al. 2014; Vieira et al. 2019).

Several factors, such as environmental conditions, hen health, hygiene practices, and egg‐handling, influence the microbial colonization of eggs throughout the production chain. Poor eggshell quality may cause damages of the eggshell, resulting in contamination of the egg with spoilage microorganisms. Consequently, this can lead to economic losses or possibly transmission of pathogens. For example, eggs laid in unsanitary environments have a higher microbial load, increasing the risk of contamination with pathogenic organisms (Chousalkar et al. 2021; Gautron et al. 2022).

In addition, environmental factors such as humidity and temperature can affect the composition of the native microflora on eggs. Although most microorganisms present in eggs are harmless or even beneficial, some pathogens, such as *Salmonella* spp., *Escherichia coli* (*E. coli)*, and *L. monocytogenes*, present serious food safety risks. It is important to understand how these pathogens behave and how prevalent they are within the egg microflora. Bacterial contamination is the leading cause of spoilage; viral contamination of egg products is rare, and contamination with yeasts and molds generally occurs during processing. Consequently, eggs are highly perishable, even when refrigerated (de Souza et al. 2015).

The effects of autochthonous microflora on the microbial shelf life of eggs or spoilage have only been investigated in a few studies to date (Chousalkar et al. 2021; Shi et al. 2020).

#### 4.1.2 Physical Defense of Eggs

**FIGURE 2:**
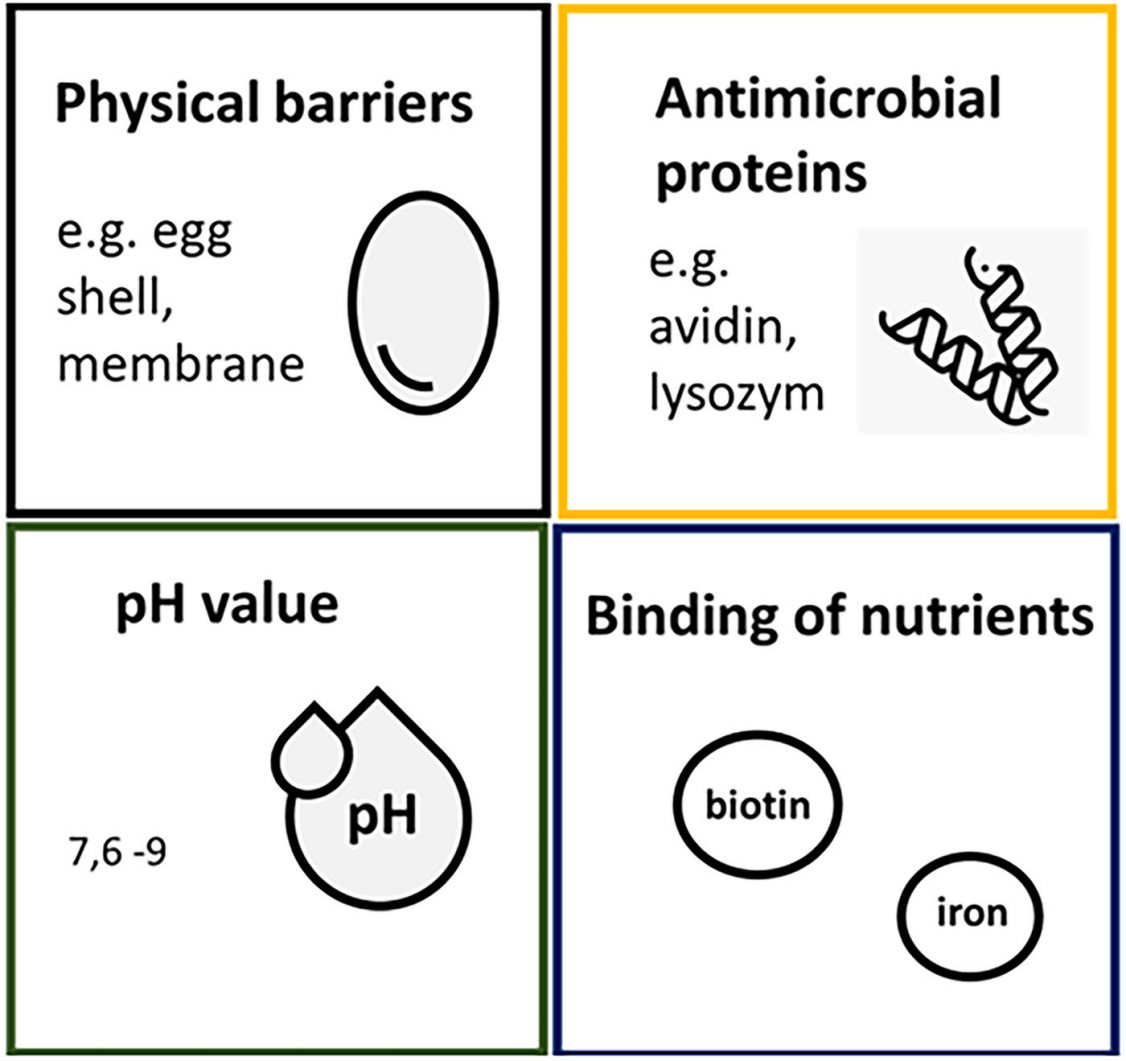
Defense mechanisms of eggs against microorganisms (own representation, based on Rathnapala et al. 2021; Feddern et al. 2017; Jabalera et al. 2022; Wengerska et al. 2023; Aygun 2017; Stefanova et al. 2021; Liao et al. 2018; Makroo et al. 2021; Kulshreshtha et al. 2018).

Eggshells and shell membranes serve as barriers against bacterial penetration into the egg (Figure 2). The quality of the eggshell is an important factor in preventing contamination of the egg content and, consequently, egg products (Chousalkar et al. 2021; Wengerska et al. 2023). The pores in the eggshell range from 9 to 35 µm in diameter, allowing microorganisms such as bacteria (0.1–6 µm), yeast (5–10 µm), and fungal spores (3–30 µm) to penetrate easily (Aygun 2017; Chen, Li, et al. 2019; Dijksterhuis 2019; Fukuda 2023; Zakhartsev and Reuss 2018). However, the cuticle blocks these pores, preventing microorganism entry into the egg. It forms the outermost physical barrier against contamination (Kulshreshtha et al. 2018; Wengerska et al. 2023). The cuticle is fragile immediately after laying, but it remains impermeable to microorganisms. Rapid chilling causes the egg to contract, pulling the cuticle into the shell pores, which can cause damages. This damage can allow microorganisms to penetrate the impermeable membrane (Kulshreshtha et al. 2018). Due to their small pore size and dense fiber structure, it is nearly impossible for microorganisms to overcome the membranes (Aygun 2017). Research by Chen, Li, et al. (2019) and Wengerska et al. (2023) has demonstrated that the quality of the membrane influences both the microbiological load and the quality. Consequently, most microorganisms enter the egg during processing steps, such as breaking (Mensah et al. 2023; Rønning et al. 2020; Torres‐Mansilla et al. 2023).

#### 4.1.3 Antimicrobial Properties of Egg White

Egg white is an inhospitable environment for microorganisms due to its antimicrobial proteins (Jabalera et al. 2022; Jalili‐Firoozinezhad et al. 2020). Furthermore, the viscosity of egg white provides another physical defense mechanism against bacteria restricting the movement. As a result, the transfer of microorganisms to the yolk, which is rich in nutrients, is hindered (Okuno et al. 2019). During egg storage, CO_2_ gradually escapes from the interior to the outside, causing the pH of the egg white to increase from about pH 7.6 to a growth‐inhibiting level for most microorganisms of around pH 9.7 (Feddern et al. 2017).

Beyond physical barriers, egg white is protected by antimicrobial proteins. Table 1 provides an overview of these proteins and their mechanisms of action. Egg white contains about 10% proteins, some of which inhibit microbial growth by binding vitamins or metal ions (ovoalbumin and ovoflavoprotein), inhibiting proteases (cysteine), or targeting the cell membrane (lysozyme) (Jabalera et al. 2022; Stefanova et al. 2021). These antimicrobial proteins are important against infections, as eggs lack immune cells (Guyot et al. 2016).

In the studies by Baron et al. (2020) and Cochet et al. (2021), the effect of exposure of *Salmonella enteritidis* (*S. enteritidis)* to egg white–based media with or without egg white proteins (>10 kDa) under bactericidal conditions (45°C) was investigated by measuring the survival rate and global expression. The authors demonstrated that the bactericidal effect of egg white at 45°C is caused not only by the proteins described below but also by small peptides in egg white that are largely responsible for its bactericidal activity.

##### 4.1.3.1 Avidin

Avidin is a glycoprotein found in egg white proteins, with a maximum concentration of 0.05% (w/w). It binds to the growth factor biotin, depriving microorganisms of an essential nutrient needed for their growth. One molecule of avidin can bind up to four molecules of biotin with very high affinity (Krkavcová et al. 2018; Li et al. 2022; Makroo et al. 2021).

##### 4.1.3.2 Cystatin

Cystatin is a protein in egg white that belongs to the family of cysteine protease inhibitors. It makes up less than 0.1% (w/w) of the total protein but has an important protective function. Cystatin specifically inhibits cysteine proteases such as papain, cathepsin B, and H, which are released by pathogenic microorganisms or the body's immune cells. By inhibiting these enzymes, cystatin helps to limit microbial virulence (Szpak et al. 2014). Additionally, cystatin is oxidatively stable and remains active under the conditions found in egg white, supporting its effectiveness in the egg's natural defense system. Recent studies suggest that cystatin has immunomodulatory properties and contribute to growth inhibition by interacting with microbial surface structures (Guérin‐Dubiard et al. 2005). Although cystatin is present in small amounts compared to other antimicrobial egg white proteins such as lysozyme or ovotransferrin, it plays a specialized role by specifically inhibiting the proteolytic attack mechanisms of pathogenic microorganisms (Szpak et al. 2014; Guérin‐Dubiard et al. 2005).

##### 4.1.3.3 Lysozyme

The important protein with antimicrobial activity is lysozyme. Making up approximately 3.5% (w/w) of egg white protein, lysozyme is the main inhibitory component of egg white (Liao et al. 2018; Shimazaki and Takahashi 2018; Stefanova et al. 2021). Lysozymes are antimicrobial enzymes, called muramidases, that catalyze the hydrolysis of the 1,4‐glycosidic bond between *N*‐acetylglucosamine and *N*‐acetylmuramic acid in bacterial cell wall peptidoglycan. Peptidoglycan gives the bacterial cell wall its strength, helping to maintain cell shape and prevent osmotic lysis. When peptidoglycan is broken down by lysozyme, the resulting osmotic imbalance causes the cell to swell and potentially burst (Liao et al. 2018; Stefanova et al. 2021). Lysozyme is primarily effective against Gram‐positive bacteria. In Gram‐negative bacteria, lipopolysaccharides coat the peptidoglycan and protect the bacteria from lysozyme's lytic activity (Liao et al. 2018; Stefanova et al. 2021). In the food industry, lysozyme (E1105) is already used as a preservative in ripened cheese (Silvetti et al. 2017).

##### 4.1.3.4 Ovoalbumin

With 54% (w/w) is Ovalbumin the main protein in egg white. Despite its abundance, ovalbumin was not originally seen as a key antimicrobial protein. This is because, unlike lysozyme or ovotransferrin, it does not directly kill bacteria. However, recent studies show that ovalbumin plays an indirect role in the antimicrobial system of egg white. It has antioxidant properties that help stabilize the egg white environment and can increase oxidative stress on microorganisms (Wang, Xu, et al. 2022). Ovalbumin can boost antimicrobial effects by interacting with other egg white proteins, such as lysozyme, by stabilizing their structure or forming networks that physically block microbial growth (Shimzaki and Takahashi 2018). Therefore, ovalbumin helps protect the embryo from microbial contamination through both direct and indirect mechanisms (Hincke et al. 2019).

##### 4.1.3.5 Ovoflavoprotein

This protein is a globular phosphoglycoprotein that comprises about 0.8%–0.9% (w/w) in egg white. It binds riboflavin (vitamin B2) with high affinity, depriving microorganisms of an essential nutrient for their growth (Liao et al. 2018; Stefanova et al. 2021).

##### 4.1.3.6 Ovoinhibitor

Ovoinhibitor makes up approximately 1%–1.5% (w/w) of total egg white. It belongs to the serine protease inhibitors and specifically inhibits enzymes such as trypsin, chymotrypsin, and elastase‐like proteases. This inhibitory effect indirectly supports antimicrobial defense, as many pathogenic microorganisms release extracellular proteases to penetrate host tissues or evade immune barriers. Inhibiting these proteases with ovoinhibitors can therefore reduce the infectious potential (Guérin‐Dubiard et al. 2005). Additionally, a study by Shimzaki and Takahashi (2018) showed that ovoinhibitors can synergistically act with other egg white proteins by enhancing the stability of the proteins and preventing proteolytic degradation.

##### 4.1.3.7 Ovomucoid

Ovomucoid is with 11% (w/w) the second most common protein in egg white, after ovalbumin. It functions as a serine protease inhibitor, mainly targeting trypsin. It plays a key role in the egg's natural defense system. The antimicrobial properties of ovomucoid primarily stem from its ability to block proteolytic enzymes produced by pathogens. By inactivating these enzymes, ovomucoid can indirectly help limit the growth of these microorganisms (Nurliyani et al. 2023; Lee et al. 2024).

Recent studies have shown that ovomucoid inhibits trypsin mainly through hydrogen bonding in its first domain region and functions as a noncompetitive inhibitor. Interestingly, applying ultrasound treatments can decrease the trypsin inhibitory activity of ovomucoid by up to 30%, indicating structural changes in the protein (Cui et al. 2024).

Furthermore, ovomucoid shows high glycosylation, which not only enhances its stability against proteolytic degradation but also affects interactions with microbial surfaces. These structural features help the protein resist thermal and enzymatic treatments.

Although ovomucoid does not have a direct bactericidal effect, it plays an important role in the egg white antimicrobial activity by inhibiting proteases. The different antimicrobial proteins, including ovomucoid, work together synergistically to inhibit the growth of pathogenic microorganisms (Nurliyani et al. 2023).

##### 4.1.3.8 Ovotransferrin

Ovotransferrin is a glycoprotein that constitutes 13% (w/w) of egg white proteins (Legros et al. 2021; Liao et al. 2018; Rathnapala et al. 2021). It binds iron‐(II)‐ions, which can be reversibly attached along with two bicarbonate ions. This process deprives microorganisms of the essential nutrient iron (Legros et al. 2021; Okuno et al. 2019; Rathnapala et al. 2021). Ovotransferrin's bacteriostatic effect is reversible with adding iron‐(II)‐ions. Once saturated, the protein can no longer bind iron, allowing microorganisms to grow (Legros et al. 2021; Liao et al. 2018).

Ovotranferrin exhibits antimicrobial activity against certain Gram‐positive and Gram‐negative bacteria and yeast strains. However, its effectiveness varies among different species. Studies showed that *E. coli*, *Pseudomonas* spp., and *Streptococcus mutans* were most inhibited by ovotransferrin, whereas *Staphylococcus aureus*, *Proteus* spp., and *Klebsiella* spp. were resistant (Rathnapala et al. 2021; Giansanti et al. 2015). This antimicrobial effect was once believed to result only from its ability to bind iron. However, Legros et al. (2021) suggest that this effect can occur independently of iron through more complex mechanisms.

Raspoet et al. (2019) demonstrated that ovotransferrin mainly causes the inhibition of *Salmonella* spp. in egg white. This only applies to a tolerance to colicin (TolC) deletion strain, not the wild‐type strain. This contradicts the results of Cochet et al. (2021), who identified two peptides that are the main bactericidal components. At this point, the precise mechanisms of inhibition by the proteins or peptides in the egg white should be investigated.

The antimicrobial activity of ovotransferrin appears to be affected by pH and temperature. Most studies focus on isolated substances, especially lysozyme, as a model enzyme against Gram‐positive bacteria. However, the impact of matrix effects on the antimicrobial properties of egg white is frequently overlooked. Only a few studies investigate the synergistic or antagonistic interactions of multiple egg white components under natural conditions (Baron et al. 2020; Legros et al. 2021).

Criticisms of the experimental conditions include the use of model solutions or artificially contaminated systems. These conditions do not accurately represent the industrial setup. Key factors, such as pH, salt concentration, protein composition, temperature, or yolk separation dilution, are rarely documented. The effects on natural microflora, particularly Gram‐negative bacteria and heat‐resistant strains, have received little attention. There is a lack of studies on potential resistance mechanisms or how microorganisms adapt to egg white inhibitors. This is a critical issue given the increasing demand for longer shelf life and alternative processing methods. To accurately evaluate their effectiveness, there is an urgent need for integrated studies that consider the interaction of various egg white components, realistic microbiological scenarios, and practical process conditions.

#### 4.1.4 Influence of pH, Storage Conditions, and Bacterial Adaptation on the Antimicrobial Activity of Egg White Proteins

##### 4.1.4.1 Influence of pH on Antimicrobial Activity

**FIGURE 3:**
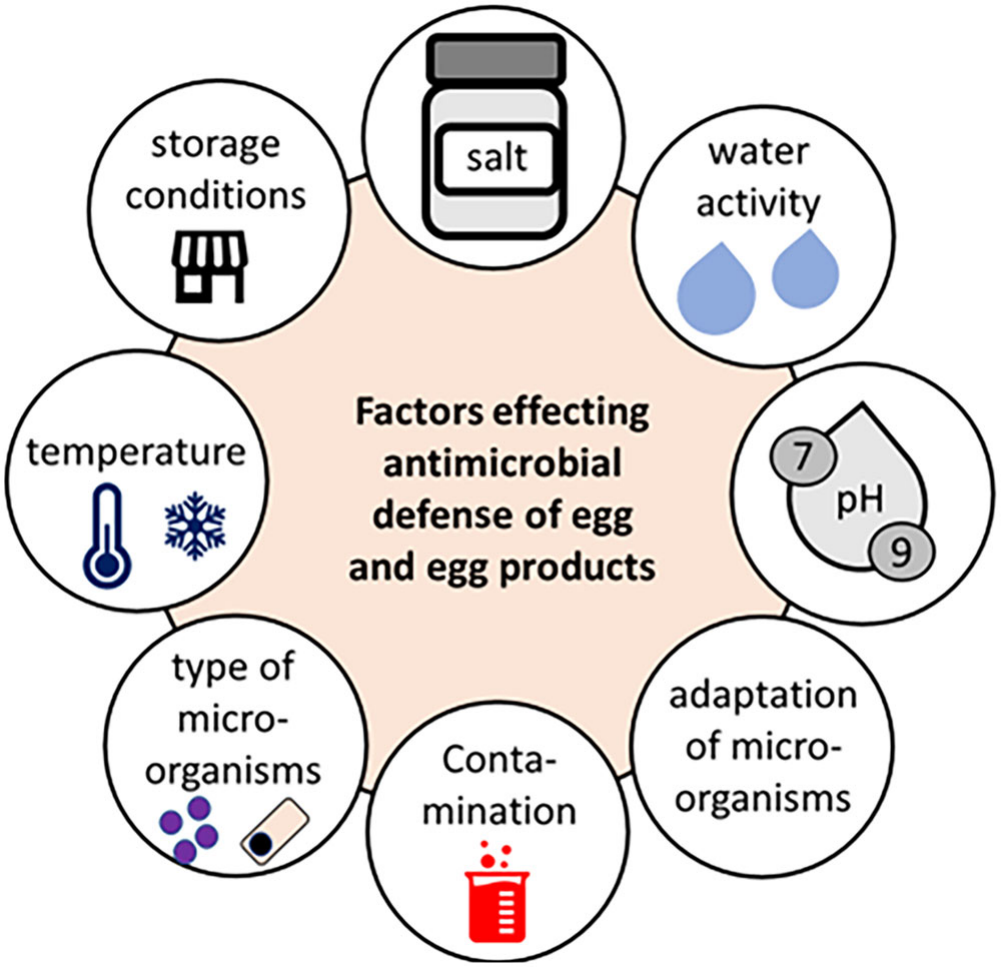
Influencing factors of the defense mechanism against microorganisms of eggs and egg products (own representation, based on Khorshidian et al. 2022; Soni et al. 2018; Qui et al. 2012; Yu et al. 2023; Liu et al. 2018; Dai et al. 2022; Legros et al. 2021; Baron et al. 2017).

Table 1 shows the antimicrobial activity of egg white proteins. Especially lysozyme, ovotransferrin, and ovomucoid are important for the natural defense of eggs against microbial contamination and have great potential for use in food safety and preservation. However, the effectiveness of these antimicrobial proteins is affected by environmental factors such as pH levels, storage conditions, and the ability of bacteria to adapt to the proteins. Figure 3 shows the factors that reduce the natural protective properties of eggs and egg white.

The pH level of egg white is a factor affecting the antimicrobial activity. Lysozyme reaches its highest activity at neutral pH (around 7). Extremely acidic conditions (pH < 4.5) or alkaline conditions (pH > 9) destabilize the protein's structure and lower its effectiveness (Khorshidian et al. 2022). This reliance on pH presents a challenge for using lysozyme in environments with fluctuating pH levels. Ovotransferrin prevents the growth of iron‐dependent bacteria such as *E. coli* and *Salmonella enterica*. In acidic conditions, ovotransferrin is most effective at releasing iron, thereby inhibiting these bacteria. However, at neutral to alkaline pH levels, this ability diminishes, reducing its antimicrobial impact (Ercan and Demirci 2016).

Ovomucoid is less pH‐sensitive than lysozyme and ovotransferrin. Extreme pH can alter the structure and, as a result, its antimicrobial activity (Legros et al. 2021). *B. cereus* spores demonstrate an inability to germinate in egg white at 4°C for 12 days, whereas cells exhibit germination at temperatures of 15°C or 25°C. When the egg white is subjected to acidification, resulting in an initial pH of 4, it has been observed that no vegetative cells are produced when stored at temperatures of 4°C, 15°C, or 25°C (Soni et al. 2018). This demonstrates that pH affects not only the structure of protein but also its antimicrobial effectiveness, showing the need for more precise pH control. Therefore, a series of hurdles should be implemented to prevent both germination and toxin production.

##### 4.1.4.2 Influence of Storage Conditions on Antimicrobial Activity

Along with pH, the storage conditions of egg white are important for maintaining their antimicrobial effectiveness. Refrigerated egg white proteins remain stable and retain their antibacterial properties, whereas higher temperatures, particularly above 20°C, cause denaturation of the proteins and a loss of their antibacterial properties (Qui et al. 2012). This temperature sensitivity impacts lysozyme, whose bactericidal effect diminishes when exposed to high temperatures, especially at alkaline pH (Khorshidian et al. 2022).

Exposure to moisture and light can diminish the antimicrobial properties of egg white proteins. High humidity often causes hydrolysis, changing the protein structure and lowering its ability to combat bacteria. The breakdown of antimicrobial peptides, especially lysozyme fragments, due to exposure to light can decrease their effectiveness (Yu et al. 2023; Liu et al. 2018; Dai et al. 2022). Egg white or its products should be stored under optimal conditions (cool, dry, and dark) to ensure long‐term use. Further study of matrix‐dependent factors (e.g., protein conformation and water activity) is crucial.

##### 4.1.4.3 Bacterial Adaptation to Antimicrobial Proteins

Egg white is a complex antimicrobial system consisting of a variety of bioactive proteins, as described in Section 4.1.3. However, the effect of these proteins is not static; it depends on bacterial adaptation processes and the physiological response of cells to the antimicrobial environment.

Bacteria such as lactic acid bacteria (LAB), *Salmonella* spp., *Listeria* spp., *Campylobacter* spp., *E. coli*, and *Staphylococcus* spp. that survive in such environments often exhibit increased expression of protective systems that stabilize the cell membrane, optimize iron acquisition pathways, or compensate for membrane damages. These mechanisms are typical of stress‐related adaptation and can reduce the antimicrobial activity of egg white proteins. Such mechanisms include peptidoglycan modifications (*N*‐deacetylation and *O*‐acetylation), changes in cell surface charge, biofilm formation, stress responses, siderophore‐mediated iron uptake, and biotin synthesis (Legros et al. 2021; Baron et al. 2017; Martínez et al. 2020). For instance, exposure to egg white can induce stress and membrane repair mechanisms in *S. enterica* serovar Enteritidis, which modulate sensitivity to ovotransferrin and other antimicrobial factors and influence the heat resistance (Legros et al. 2021; Baron et al. 2016, 2017, 2020; Huang et al. 2019).

The adaptation of bacteria to the antimicrobial proteins in egg white should primarily be understood as a physiological and regulatory response to a stressful environment. For the egg industry, this means that although the antimicrobial effect of egg white is not completely lost, its effectiveness can decrease (Jabalera et al. 2022; Baron et al. 2020; Legros et al. 2021). From a technological perspective, it is problematic that adapted bacteria can exhibit increased survivability under production and storage conditions. This applies to both spoilage organisms and potential pathogens that can persist in liquid egg white or egg‐containing products. Adaptation can also lead to cross‐protection effects in which the stress response, induced by egg white proteins, simultaneously increases tolerance to other stresses such as heat treatment. This can reduce the effectiveness of process steps (Khan et al. 2021; Xu et al. 2022; Baron et al. 2017, 2020). From an industrial standpoint, this suggests that process parameters should be selected in a way that results in either direct sufficient inactivation or the reduction of stress phases. In order to counteract bacterial adaptation in industry, combined hurdle strategies (also known as hurdle technology) are utilized. In this context, the use of nonthermal methods is also gaining importance. It has been shown that high‐hydrostatic‐pressure (HPP) treatment, pulsed electric fields (PEFs), ultrasound (US), or combinations can damage cell membranes and thus counteract bacterial adaptation mechanisms without damaging the functional properties of egg white. These methods act in a synergistic manner with natural antimicrobial proteins (Baron et al. 2017; Xia et al. 2022). By controlling environmental conditions and understanding bacterial adaptation, egg white proteins can be used more effectively in the food industry and beyond.

### 4.2 Extrinsic Factors

#### 4.2.1 Bacterial Contamination

The housing system for hens affects the level of microbiological contamination of eggs. Eggs from caged hens are less microbiologically contaminated than eggs from alternative housing systems (Holt 2021). In their study, Parisi et al. (2015) were able to show that, for example, 26.1% of free‐range egg samples were contaminated with *Campylobacter* spp. and 2.36% with *Salmonella* spp., in contrast to eggs from caged hens, which were free of *Salmonella* spp. Contamination, and only 7.4% of samples were contaminated with *Campylobacter* spp. The native microbiota of eggshells mainly includes Gram‐positive bacteria such as *Staphylococcus* spp., *Streptococcus* spp., *Aerococcus* spp., *Bacillus* spp., and *Micrococcus* spp., along with some Gram‐negative bacteria such as *Campylobacter* spp., *Salmonella* spp., *Escherichia* spp., and *Alcaligenes* spp. (Aygun 2017; Techer et al. 2014; EFSA 2014a; Tomczyk et al. 2018). Table 2 offers an overview of common microorganisms found on eggs and in egg products.

**TABLE 2:** Microorganisms and viruses on eggs and in egg products (+ = positive; − = negative; na = not applicable; ni = not indicated).

Genre of microorganism	Microorganism type	Location of contamination	Place of origin	Type of spoilage	Temperature tolerance	Respiration	Gram stain	References
*Achromobacter*	Bacteria	Eggshell, egg content	Soil, water	Rotten eggs	ni	Aerobic	−	Busse and Auling (2015)
*Acinetobacter*	Bacteria	ni	Soil, water	Rotten egg with odors and color change	ni	Aerobic	−	Nemec (2015)
*Aerococcus*	Bacteria	Eggshell	Air, soil, water	Human pathogen	ni	Facultative anaerobic	+	Techer et al. (2014); Collins and Fallsen (2015)
*Aeromonas*	Bacteria	Eggshell, egg content	Water	Rotten eggs, gastroenteritis, sepsis	ni	Facultative anaerobic	−	Aygun et al. (2017)
*Alcaligenes*	Bacteria	Eggshell	Water, soil	Facultative pathogens, rotten egg with odors and color change	Psychrotolerant	Obligate aerobic	−	Aygun et al. (2017), Techer et al. (2014)
*Arthrobacter*	Bacteria	Eggshell	Soil	ni	Low temperatures	Aerobic	+	Aygun et al. (2017)
*Bacillus*	Bacteria	Eggshell	Environment	Pathogen linked to foodborne outbreaks	Heat resistant spores	Facultative aerobic	+	Aygun et al. (2017), Techer et al. (2014)
*Campylobacter*	Bacteria	Eggshell	ni	Pathogen linked to foodborne outbreaks, diarrhea, belongs to the zoonotic pathogens, mandatory for reporting	no	Microaerophilic	−	Aygun et al. (2017)
*Citrobacter*	Bacteria	Eggshell	Gut flora human	Pathogen linked to foodborne outbreaks, rotten egg with odors and color change	ni	Facultative anaerobic	−	Aygun et al. (2017)
*Enterobacter*	Bacteria	ni	ni	Rotten egg with odors and color change	ni	Facultative anaerobic	−	Aygun et al. (2017), Coutinho et al. (2015)
*Escherichia*	Bacteria	Eggshell	Gut flora from a bird's mammalian	Pathogen linked to foodborne outbreaks, rotten eggs with odors, and color change	Can survive pasteurization	Facultative anaerobic	−	Aygun et al. (2017), Techer et al. (2014)
*Listeria*	Bacteria	Eggshell	Ubiqitary	Pathogen linked to foodborne outbreaks	Psychrotolerant	Facultative anaerobic	**+**	Aygun et al. (2017)
*Micrococus*	Bacteria	Eggshell	Ubiqitary		ni	Aerobic	**+**	Aygun et al. (2017)
*Moraxella*	Bacteria	ni	ni	Rotten egg with odors and color change	ni	Aerobic	−	Aygun et al. (2017), Fernández‐Garayzábal et al. (2015)
*Salmonella*	Bacteria	Eggshell	Mammalian	Pathogen linked to foodborne outbreaks, reportable	ni	Facultative anaerobic	−	Aygun et al. (2017), Techer et al. (2014)
*Serratia*	Bacteria	Eggshell	Water, soil, animals, and plants	Mostly a pathogen for humans, rotten eggs with odors and color change	ni	Facultative anaerobic	−	Aygun et al. (2017)
*Staphylococcus*	Bacteria	Eggshell	Human and animal skin	Pathogen linked to foodborne outbreaks	Survive 20 min at 80°C	Facultative anaerobic	**+**	Aygun et al. (2017), Montanari et al. (2015), Techer et al. (2014)
*Proteus*	Bacteria	Eggshell	Humans	Facultative pathogen, rotten egg, with odors and color change	ni	Facultative anaerobic	−	Aygun et al. (2017)
*Pseudomonas*	Bacteria	Eggshell	ni	Rotten egg with odors and color change	ni	Or anaerobic	−	Techer et al. (2014), Mansour et al. (2015)
*Alternaria*	Mold	Eggshell, less egg content	ni	Mycotoxins	ni	Aerobic	na	Tomczyk et al. (2018), Tomczyk et al. (2019), Haque et al. (2020), Gai et al. (2023)
*Cladosporum*	Mold	Eggshell, less egg content		ni	ni	Aerobic	na	Tomczyk et al. (2018), Tomczyk et al. (2019), Haque et al. (2020)
*Candida*	Yeast	ni		Facultative pathogens	ni	Aerobic	na	Haque et al. (2020), Tomczyk et al. (2018), Tomczyk et al. (2019)
*Chaetomian*	Mold	Eggshell		ni	ni	Aerobic	na	Haque et al. (2020), Tomczyk et al. (2018), Tomczyk et al. (2019)
*Fusarium*	Mold	Eggshell, less egg content	Mycotoxins	ni	ni	Aerobic	na	Haque et al. (2020), Tomczyk et al. (2018), Tomczyk et al. (2019)
*Mucor*	Mold	Eggshell, less egg content	Mucormycoses	ni	ni	Aerobic	na	Tomczyk et al. (2018), Tomczyk et al. (2019), Haque et al. (2020)
*Penicillium*	Mold	Eggshell, less egg content		ni	ni	Aerobic	na	Tomczyk et al. (2018), Tomczyk et al. (2019), Haque et al. (2020)
*Trichoderma*	Mold	Soil		ni	ni	Aerobic	na	Haque et al. (2020), Tomczyk et al. (2018), Tomczyk et al. (2019)
Avian Influenza (AI)	Virus	Hen		Drop in egg production up to 100%; mishappen, discolored, and fragile eggs	ni	na	na	Hassan and Abdul‐Careem (2020)
Avian hepatitis E (HEV)	Virus	Hen		Drop in egg production	ni	na	na	Gonzales et al. (2018), Sun et al. (2019)
Newcastle disease (ND)	Virus	Hen		Drop in egg production; decreased shell thickness, soft shells, spotted shells, and decreased egg white height	ni	na	na	Han et al. (2017)
Infectious bronchitis (IB)	Virus	Hen		Drop in egg production up; misshapen eggs, rough and thin shells, eggshell discoloration, and watery egg white	ni	na	na	Abozeid (2023), Rafique et al. (2024)
Egg drop syndrome (EDS)	Virus	Hen		Drop in egg production; pale eggshell, thin, soft, and shell. Less eggs, watery thin egg white	ni	na	na	Al‐Ebshahy et al. (2024)

Microbial contamination can cause hygiene issues and spoilage, potentially leading to foodborne illness outbreaks and economic losses (Sarno et al. 2021; Techer et al. 2019). The most common pathogen found in and on raw egg products is *Salmonella* spp. (Techer et al. 2019). The hygienic concern mainly involves *S. enteritidis*, one of the key pathogens linked to outbreaks associated with eggs and egg products. This microorganism can form biofilms on the eggshell and penetrate the pores (Lin et al. 2020). It can contaminate both the eggshell and the interior of the eggs, including the yolk and egg white. According to a study by Gast et al. (2014), *S. enteritidis* can survive on eggshells for long periods, making proper handling and preparation fundamental to prevent infection.

Once eggs are cracked, they lose their antimicrobial properties and become vulnerable to contamination. Egg products can be contaminated not only through vertical or horizontal transmission but also via all usual routes of contamination that affect most foods, such as cross‐contamination during manufacturing, as mentioned earlier, and transmission of pathogens from other infected or contaminated foods, from handlers, contaminated equipment, or biofilms (Borges et al. 2018; Lee et al. 2020; Sharaf Eddin et al. 2019).

*L. monocytogenes* can form persistent biofilms on equipment surfaces, leading to intermittent contamination of products during processing (Finn et al. 2023; van de Merwe et al. 2024). The conditions used in industrial processing and pasteurization of eggs, along with the dilution of microorganisms when many eggs are combined, are generally effective. However, process failures can occur, resulting in outbreaks of food poisoning linked to processed liquid egg products (EFSA 2014a, 2014b). In 2021, eggs and egg products were the second most common source of foodborne outbreaks in the EU, with *Salmonella* spp. being the main pathogen (EFSA 2022).

The European Food Safety Authority (EFSA) report (2014a, 2014b) provides detailed information on the relatively few bacterial outbreaks caused by pathogens other than *S. enteritidis*. The main pathogens include *B. cereus*, *Campylobacter jejuni* (*C. jejuni*), *E. coli*, and *S. aureus* (EFSA 2014a, 2014b). However, the role of egg products in human infections with these bacteria remains unclear, and cross‐contamination from other foods and/or equipment is an important factor.

Most studies concentrate on classic foodborne pathogens, such as *C. jejuni*, *S. enterica*, *L. monocytogenes*, and certain Enterobacteriaceae. However, the broader microbial spectrum has received less attention. There is limited current information on microbial contamination in dried egg white. Some heat‐resistant organisms that survive the pasteurization of liquid eggs persist through the drying process. Therefore, it is possible for the dried egg to become contaminated afterward (Wei et al. 2020).

Although conventional cultivation methods and molecular biological techniques are applied, they are not used in a standardized manner. For the determination of *Salmonella* spp., for example, Gast et al. (2014) used cotton swabs for sampling, which are incubated in tetrathionate broth for 24 h at 37°C and then spread on brilliant green agar. In contrast, Techer et al. (2019) used metal chips for sampling, which were suspended in peptone water and spread directly onto Brain Heart Infusion agar. This methodological heterogeneity contributes to an overall fragmented data situation, preventing a reliable assessment of the typical microbial composition of egg white under industrial conditions. A particular gap in the research lies in the lack of metagenome analyses that are sufficiently comprehensive to detect microorganisms that cannot be cultivated or are currently unknown.

##### 4.2.1.1 Mechanism of Bacterial Contamination of Eggs

**FIGURE 4:**
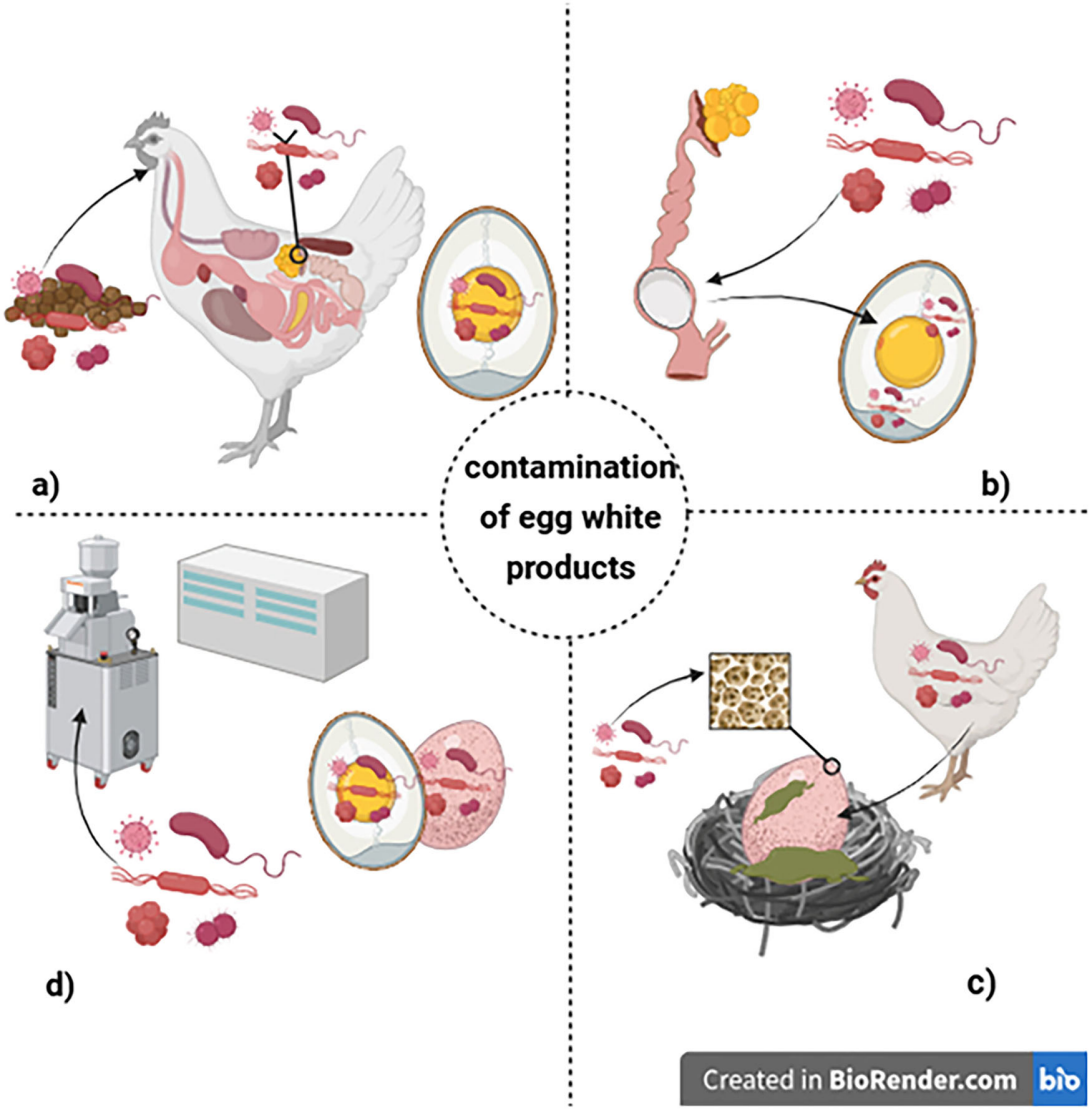
Pathways of contamination of egg white products. Potential sources of microbial contamination along the production chain of hens’ eggs are shown: (a) vertical transmission: transovarian infection—microbial entry into the egg during follicle formation in the ovary; (b) vertical transmission: oviductal infection—contamination of the egg contents by microorganisms during passage through the oviduct; (c) horizontal transmission: trans‐shell‐infection—post‐ovipositional bacterial entry through the shell, favored by pores, cracks, or moisture; and (d) horizontal transmission: contamination when breaking the eggs—secondary contamination of the egg white during industrial processing, especially in the case of poor hygiene when the eggs are opened by machine (own representation created in https://BioRender.com, based on Aygün and Narinç 2017; Cafarchia et al. 2019; Chousalkar et al. 2021; Lee et al. 2020; Liu et al. 2023; Techer et al. 2019).

These microorganisms can contaminate eggs and egg products in four ways: through vertical transmission (transovarian infection or oviduct infection), horizontal transmission, or processing steps in the industry. Figure 4 provides a schematic overview of these transmission routes. The route of transmission depends on how the microorganism contacts the egg (Techer et al. 2014; Chousalkar et al. 2021). Therefore, microbial contamination of eggs occurs either from the environment or systemically through infected chickens or their environment. Vertical or systemic contamination happens when chickens ingest feces from other infected chickens during feeding, leading to systemic infection in the chicken's gut (Neira et al. 2017).

Vertical transmission (Figure 4a,b) involves the direct transfer of pathogens from the parent to the egg interior, especially through infections of the reproductive tract of laying hens. This transmission mode is especially important for pathogenic bacteria such as *S. enterica* serovar Enteritidis. Vertical transmission typically occurs via two mechanisms. During the transovarian infection (Figure 4a), the pathogen enters the ovarian follicle through a systemic infection of the hen (often hematogenous) that occurs before ovulation.

The second mechanism involves oviduct infection (Figure 4b). After ovulation, the egg white or the eggshell membrane can become contaminated during its passage through the oviduct if there is a local infection, such as with *S. enteritidis*. In this case, contamination happens before the formation of the calcified shell, meaning that cleaning the shell afterward does not remove the pathogen inside the egg.

The most common pathogen in vertical transmission is *Salmonella* spp. (Chousalkar et al. 2021; Liu et al. 2023). For example, *S. enteritidis* can enter the chicken's bloodstream and transovarially infect the egg follicle, leading to yolk contamination, which can eventually pass into the egg white (transovarian infection). Another way eggs can become contaminated through vertical infection is via oviductal infection, where the egg gets contaminated during its passage through the chicken's oviduct. Pathogens enter the oviduct either through the blood system or by ascending from the cloaca (Chousalkar et al. 2021; Liu et al. 2023; Aygün and Narinç 2017).

In horizontal transmission (Figure 4c), the eggs become contaminated after laying by contact with contaminated surfaces, such as dirty nests, feces, dust, containers, and other animals. The microorganisms can enter the egg through tiny cracks or pores in the shell, spoiling the egg white (trans‐shell infection) (Aygün and Narinç 2017).

Egg contents inside shells are usually uncontaminated right after laying, although vertical contamination can happen in some cases. Egg whites are usually contaminated with microorganisms during processing or storage. Therefore, contamination consistently occurs during egg processing, either through contact of eggshells with egg contents during breaking or from contact with contaminated surfaces (Figure 4d) (Cafarchia et al. 2019; Lee et al. 2020; Techer et al. 2019; Yin et al. 2018).

#### 4.2.2 Viral Contamination

The risk of virus transmission over eggs to consumers is very low because the contents of eggs do not create an ideal environment for foodborne viruses to grow (Bosch et al. 2018; Gonzales et al. 2018).

Some known cases exist where zoonotic viruses, such as avian influenza and avian hepatitis E virus, have been transmitted to humans (Bosch et al. 2018; Gonzales et al. 2018; Sun et al. 2019). However, there is currently no evidence indicating that the viruses were transmitted through food. It has been suggested that environmental exposure might be the source (Horm et al. 2016; Li et al. 2014). Transmission of the virus from egg whites to humans may be possible but appears unlikely, especially because infected eggs do not enter the market.

The infection in laying hens mainly reduces egg production, makes the eggshell thinner and more vulnerable to damage, and decreases egg quality. These eggs usually do not make it to the market and are destroyed (Hassan and Abdul‐Careem 2020; Liu et al. 2017).

Although most studies focus on the vertical transmission of avian viruses, data on the actual transfer of these viruses into the egg are rare. There is little known about the risk of virus transmission to humans through the consumption or handling of contaminated eggs, which makes comprehensive risk assessments difficult. The use of non‐standardized detection methods, different virus strains, and artificial inoculation models limits the comparability of existing data further.

#### 4.2.3 Fungal Contaminations

To date, only a limited number of studies have examined egg and egg product contamination with fungi (Tomczyk et al. 2018, 2019). Various fungal genera such as *Penicillium* spp., *Alternaria* spp., *Chaetomium* spp., *Cladosporium* spp., *Fusarium* spp., *Stemphylium* spp., *Mucor* spp., *Trichoderma* spp., and *Rhizopus* spp. have been identified on the eggshell (Haque et al. 2020; Tomczyk et al. 2018, 2019). Table 2 offers an overview of the types of fungi found on eggs.

Contaminations with fungi happen during improper storage (Tomczyk et al. 2018). Egg storage temperature, fungal cell density, and fungal size may influence eggshell penetration. For example, mold hyphae (1–30 µm) can penetrate the eggshell through its pores (9–35 µm) and contaminate the egg white (Aygün and Narinç 2017; Cafarchia et al. 2019; Islam et al. 2017).

Generally, fungi cause more spoilage than foodborne illness outbreaks. Because of high humidity and poor air circulation, frozen egg products are often contaminated with molds (Haque et al. 2020).

Cafarchia et al. (2019) examined eggs for the presence of yeasts and found mainly *Candida* spp. within the egg content. These are the natural gut microflora of chickens that usually do not cause foodborne disease outbreaks but can be opportunistically pathogenic (Cafarchia et al. 2019).

Various molds, such as *Aspergillus* spp., *Fusarium* spp., and *Penicilium* spp., can produce mycotoxins that are harmful to humans. Carry‐over of mycotoxins into eggs seems unlikely, as they rarely pass into eggs but tend to accumulate in tissues. In the study by Tomczyk et al. (2019), *Fusarium* spp. mycotoxins were detected in egg white. Fungal invasion and the presence of mycotoxins in this study were linked to housing conditions and moisture rather than transmission through feed. As the practices of floor and free‐range farming become increasingly common, there is an increased possibility of exposure to temperature fluctuations and environmental contamination, which can stimulate fungal growth. Contamination of eggs with mycotoxins via the hens has not been confirmed. Egg products are more prone to contamination by mold due to suboptimal storage conditions (temperature and humidity), which can result in the formation of mycotoxins (Wang et al. 2015; Wang, Zhang, et al. 2018).

#### 4.2.4 Probiotics

##### 4.2.4.1 Influence of Probiotics on Hens’ Health

Antibiotics have been used in the poultry industry for many years. They have two functions: preventing diseases and promoting growth. However, this has led to an increase in antibiotic‐resistant bacteria, which are a threat to both animal and human health. The European Union (EU) banned the use of antibiotics as growth promoters in animals in 2006. The US Food and Drug Administration (FDA) prohibited the use of antibiotics for humans in livestock farming in 2017 (Rahman et al. 2022).

Better hygiene practices, alternative prevention methods, and more focus on animal healthcare are needed. Research has shifted to studying the microbiota of chickens and how it affects egg quality and products. Probiotics have an indirect effect on egg white quality by improving hen gut health and nutrient absorption, boosting the immune system, and lowering microbial risks. This may influence the physicochemical and microbiological properties of egg whites (Balta et al. 2022; Qiu et al. 2022; Salem et al. 2023; Ahmad et al. 2022).

Probiotics are a method to improve the quality and microbiological safety of eggs and egg products. Lactobacilli are among the most common bacteria, which mainly enhance the gut microbiome, thereby positively impacting chicken health (Ranjha et al. 2021; Ricke et al. 2022). Specifically, after illness, animals recover more quickly and develop greater resistance (Khan and Chousalkar 2020; Wang, Li, et al. 2018).

The effectiveness of probiotics to improve immune function (Fathi et al. 2018), gut health (Khan and Chousalkar 2020; Yan et al. 2019; Yang et al. 2020), productivity (Hosseindoust et al. 2018; Ricke et al. 2022; Yan et al. 2019), and egg quality, especially the quality of the shell and egg white (Chung et al. 2014; Neijat et al. 2019; Song et al. 2019), has already been demonstrated in numerous studies. This topic is discussed in greater detail by Jha et al. (2020) and Al‐Otaibi et al. (2023).

##### 4.2.4.2 Influence of Probiotics on Egg Safety and Quality

Other studies have shown that feeding probiotics can increase egg resistance to pathogens by strengthening eggshells and improving protein quality (Gan et al. 2020; Neijat et al. 2019; Song et al. 2019; Zhan et al. 2019).

The transmission of *C. jejuni* was effectively reduced by adding probiotic bacteria to the feed (Balta et al. 2022). Saint‑Cyr et al. (2017) showed in their study that administering 10^7^ colony‐forming units (CFU) of *Lactobacillus salivarius* (SMXD51) reduced *Campylobacter* contamination in broilers by 2.81 log. A study by Wang, Li, et al. (2018) demonstrated that *Lactobacillus plantarum* (*L. plantarum)* can protect the host from intestinal barrier disruption caused by *Salmonella* spp. infection. Probiotics for chickens are used commercially, with the aim of promoting their overall health and well‐being.

The positive effects of probiotics can not only enhance the quality of eggs and egg whites but inhibit the growth of harmful bacteria (Ben Lagha et al. 2017; Ding et al. 2021). The butyric acid produced by many probiotic bacteria acts as an energy source for epithelial cells, which serve as a barrier against pathogenic organisms (Banasiewicz et al. 2020; Elnesr et al. 2020). A study by Redweik et al. (2020) confirmed the potential of probiotics to reduce pathogen infections in chickens. The study examined resistance to *Salmonella* Kentucky (CVM29188) in a strain feed test. The control group and the vaccinated group had the highest number of CFU per gram (g) of feces, at 10^4^ CFU/g. The probiotic group had a lower number at 10^3^ CFU/g feces, and no *Salmonella* was detected in the feces of the group that received both vaccination and probiotics. It is known that some LAB, such as *Lactobacillus rhamnosus*, *L. plantarum*, or *Lactobacillus paracasei*, produce antimicrobial substances that can inhibit fungi like *Aspergillus* spp., *Candida* spp., and *Fusarium* spp. (El‐Ashmony et al. 2022), viruses such as influenza, and Newcastle disease (Abdelhamid et al. 2019), and other pathogens, including *Bacillus* spp., Enterobacteriaceae, *Salmonella* spp., and *Clostridium* spp. (Lin and Pan 2019; Olnood et al. 2015). Using probiotics to improve the microbiological safety and quality of chicken eggs and egg products while reducing antibiotic use is a promising approach that deserves further research (Ben Lagha et al. 2017; Qiu et al. 2022; Zhao et al. 2019).

Understanding how feed additives interact with the functional properties of egg whites could offer new insights into their effects on laying hens and provide an alternative way to modify the functional properties of egg whites.

##### 4.2.4.3 Effective Microorganisms (EMs) for Enhancing Egg Quality

EMs include various microorganisms, such as photosynthetic bacteria, actinomycetes, lactobacilli, and fungi (Ezeagu et al. 2023). They can be added to chicken feed and water to enhance intestinal flora, improve digestion, and reduce feed costs (Gnanadesigan et al. 2014). This boosts profitability. Adding EM to a hen's diet improves resistance to illness and performance (Xiang et al. 2019, Dehsahraee et al. 2024). The study by Atsbeha and Hailu (2021) shows that adding EM to feed and water could increase daily egg production. The average daily egg production was 18.42 ± 1.2 for chickens fed with EM in water and feed compared to 11.9 ± 1.1 in the control group. The quality of the eggs was also enhanced by feeding EM, as evidenced by increased eggshell thickness around ∼0.058 mm and egg white weight ∼2.53 g, in comparison to the control group.

From our perspective, using probiotics and EM can reduce the need for antibiotics and meet the rising consumer demand for “natural” and “antibiotic‐free” products. Standardizing the dose and application method and demonstrating functional efficacy in production systems are challenges that must be addressed.

## 5 Egg White Technology and Effect on Functionality

### 5.1 Background in the Egg White Technology

Egg white quality is influenced during processing, leading to a need for new technologies to produce safe, high‐quality products. These methods improve safety and shelf life, but not all are approved for treating egg whites. Egg whites can be sensitive to thermal treatment, especially at higher temperatures, which can damage the protein structure or functional properties. Proteins can be prevented from thermal degradation by using nonthermal methods, which involve the use of radiation or other techniques and lower temperatures.

**FIGURE 5:**
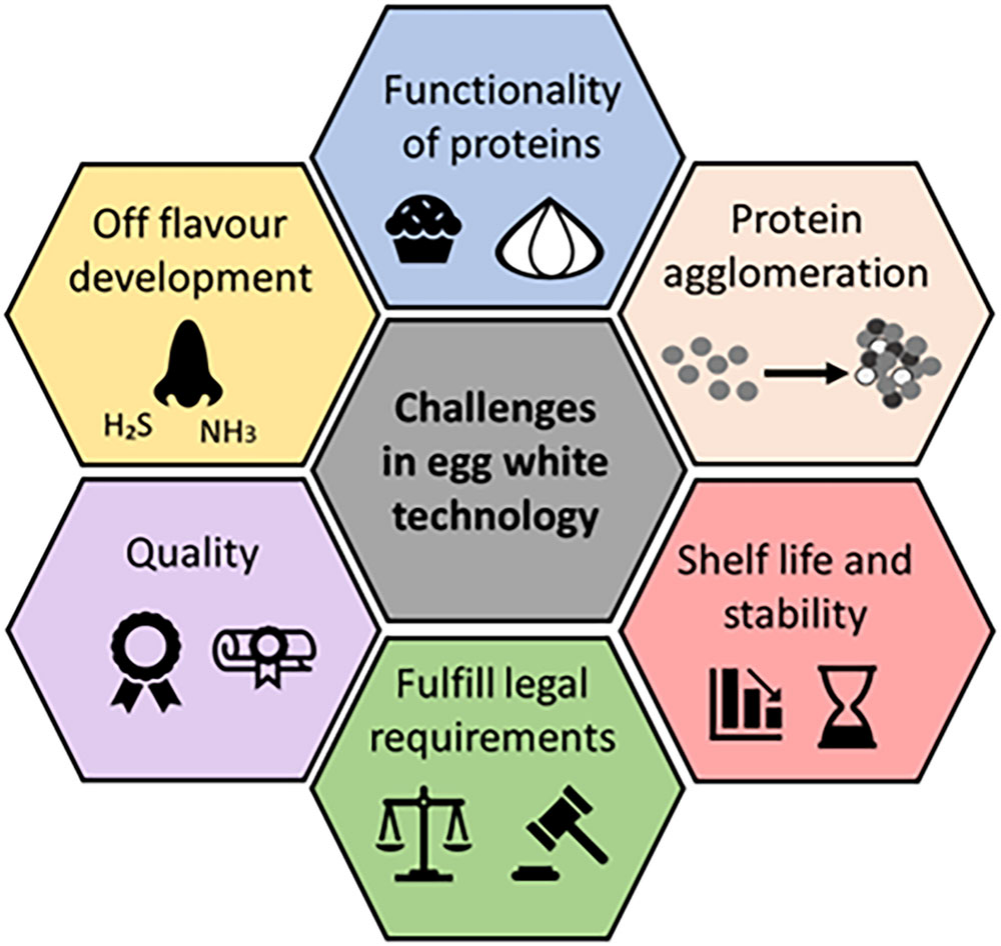
Overview of current challenges in egg white technology (own representation, based on Afraz et al. 2020; Bhat et al. 2021; Liu et al. 2019; Fan et al. 2024; Razi et al. 2023).

These approaches enhance product quality, modify functional properties, and extend shelf life, particularly with regard to bacteria's resistance to the antimicrobial proteins in egg white. When developing new egg white‐based products through fermentation using pathogen‐free raw materials is critical for safe products. Figure 5 shows pasteurizing egg white involves challenges.

### 5.2 Effect of Raw Material

Several factors affect raw egg quality, including breed, nutrition, health status, age, storage duration/temperature, pH, and initial bacterial load, as well as time before cracking. The quality of the egg white is influenced both during its formation in the hen and after the egg is laid. As hen age increases, egg white becomes liquid, and the gel structure weakens. Eggs are aging immediately after laying, and their chemical, functional, microbiological, and physical properties change (Alamprese et al. 2012; Mudannayaka et al. 2019).

Oxidative stress negatively impacts egg white quality, reducing its taste, odor, antioxidant properties, and nutritional value (Duan et al. 2018). Egg odor can be influenced by oxidative stress, the type of feed given to the chickens, and the presence of microorganisms. The most studied egg odor is fish, which can enter the egg through feed containing fish meal, soy meal, or canola. This scent is caused by the substance trimethylamine (TMA). In chickens with a genetic defect, TMA oxidase is inhibited in the liver, allowing TMA to enter the egg. This off‐flavor mechanism is known in commercial farms and is easily managed (Feng et al. 2020). Studies indicate that the laying farms (organic, cage, or free‐range) and hen age do not affect the functional properties of the egg (Filipiak–Florkiewicz et al. 2017; Vlčková et al. 2019). Hens at the end of their laying cycle produce more eggs with fragile, porous shells, which impact the physicochemical properties of the egg white and lower the barrier for microorganism (Park and Sohn 2018).

The factors influencing quality after laying depend on storage duration, temperature, and the age of the hens. Freshly laid chicken eggs have a pH of 7, but as they lose carbon dioxide (CO_2_) through the shell, the pH rises to an alkaline level of up to 9 (Luo et al. 2020). This makes the egg white more transparent and watery and weakens the yolk membrane, which can cause the yolk to mix into the egg white when beaten, reducing quality. Higher storage temperatures (25°C and 35°C) accelerate this process, further decreasing the quality of the egg white (Luo et al. 2020). As the pH of the egg white increases during storage, some of the protein converts from *n*‐ovalbumin to heat‐stable *s*‐ovalbumin, which is less hydrophobic than *n*‐ovalbumin. This change affects the functional properties of the egg white, especially its ability to form foams. The property of creating a cohesive film at the air–water interface diminishes, leading to reduced foam stability (20.16% after 40 days of storage) and increased water outflow (Chen, Sheng, et al. 2019b; Deleu et al. 2015).

Microbiological contamination is another important factor influencing the quality and safety of egg products. Due to contamination in the environment, high initial microorganism load from ∼10^4^ to 10^5^ colony forming units (CFU/mL) can limit the effectiveness of preservation methods (see Sections 5.3 and 5.4). The higher the initial bacterial content, the greater the possibility that individual microorganisms will survive the pasteurization and grow during storage (Techer et al. 2015; Baron et al. 2020; Soni et al. 2022). For example, *Salmonella* spp. has a higher heat tolerance at alkaline pH levels and can survive pasteurization (Kim et al. 2019). As storage time increases, the pH changes and the egg proteins are modified, which can negatively affect antimicrobial activity, foam formation, gelation, and heat stability (Yu et al. 2023; Rehault‐Godbert et al. 2010). The age of laying hens affects their protein composition. Hens of 52 weeks (>20%) have higher concentrations of ovotransferrin than those of 25 weeks (<20%) (Jabalera et al. 2022). These aspects illustrate that the microbiological quality of protein is closely linked to raw material quality, storage conditions, and processing steps.

### 5.3 Traditional Methods of Egg White Technology

#### 5.3.1 Pretreatment of Eggs

In the sorting center, eggs are sorted to determine their destination. Groups M and L are the two most commonly sold because they meet consumer demand. The other groups are mainly used for the production of egg products. Eggs with a defective shell may be used for making egg white products but must be processed as soon as possible (Gautron et al. 2021; Nys et al. 2018).

After sorting, eggs are typically transported to an egg processing plant. There, they are usually washed and/or disinfected to remove contaminants from the shell. However, this process is not permitted in all countries, especially in Europe. In the present review, only the processing of egg white will be discussed in more detail. The egg white is filtered to remove shell residues before further processing (Mattisek and Fischer 2021).

#### 5.3.2 Conventional Thermal Treatments

Egg content is prone to microbial contamination after breaking and must be pasteurized quickly. Egg whites are pasteurized at temperatures between 56°C and 58°C for 2–3 min. In international comparisons, information about temperatures and holding times varies (Bermudez‐Aguirre and Niemira 2023; Gautron et al. 2021, 2022). The USDA recommends pasteurization at 57°C for 3.5 min as *S. enteritidis* is much more sensitive at alkaline pH (8.0–9.5). For lower pH levels (∼7), a temperature of 60°C is suggested (USDA 2023). A study by Lechevalier et al. (2017) demonstrated that even a few degrees higher in temperature (60°C, 62°C, and 66°C) or a few more minutes (4, 7, and 10 min) of treatment can change the quality and functionality of the egg product, such as decreasing the coagulation and foaming properties of egg white. Most proteins are stable within this temperature range and only denature above the pasteurization temperature (Table 1). Notably, antimicrobial proteins like ovotransferrin and lysozyme in egg white denature above the pasteurization temperature and help maintain microbial stability during storage (Table 1) (Bermudez‐Aguirre and Niemira 2022; Legros et al. 2021). Despite the relatively low pasteurization temperature below 60°C, the proteins responsible for functional properties, such as ovalbumin and conalbumin, get damaged. This usually leads to a loss of functional properties such as foam stability (Gharbi and Labbafi 2018; Razi et al. 2023). The foam stability of pasteurized egg whites can be improved by using aluminum sulfate (E520) or hydrocolloids as additives (soybean hemicellulose E426) (European Commission 2011). The aluminum salts prevent denaturation of the egg white's most temperature‐sensitive protein, conalbumin. Hydrocolloids, on the other hand, bind the free water, which prevents water from escaping from foam lamellae in foamed egg white (Pirsa and Hafezi 2023; Razi et al. 2023).

#### 5.3.3 Treatment by Cooling and Freezing

After pasteurization, the egg white can be chilled or frozen for longer storage. Fresh, pasteurized liquid egg white can be stored at a temperature of 4°C in sealed packaging for a period of 7–10 days. After this period, protein degradation, viscosity loss, and functional decline occur, in the case of heat‐sensitive components such as ovomucin and ovotransferrin.

Freezing egg white is a common way to extend its shelf life, especially in the food industry and during further processing of egg products. At −18°C, liquid egg white can be stored for up to 12 months without becoming microbiologically unsafe (Guo et al. 2022). However, some studies show that long‐term freezing, even at steady temperatures, causes functional and structural changes in the main proteins of egg white. Despite being relatively heat‐stable, ovalbumin remains vulnerable to structural changes, which can decrease its solubility (Yu et al. 2023; Stănciuc et al. 2016). Ovotransferrin and ovomucin are more sensitive to freezing stress: ovotransferrin can lose its metal‐binding ability, while ovomucin, which helps give fresh egg white its gel‐like texture, is destabilized by ice crystal formation in its network. This can reduce viscosity and impair foam formation and stability (Wang, Gu, et al. 2020a). Repeated freezing and thawing are especially problematic because they cause irreversible aggregation and exposure of hydrophobic amino acids (Zhang, Zhu et al. 2023). Studies indicate that rapid freezing (e.g., below −30°C with fast cooling) better preserves protein structure than slow freezing, as it produces smaller ice crystals that cause less damage to cells and proteins. To minimize quality loss, industrial settings often use shock‐freezers for quick freezing (James et al. 2015).

#### 5.3.4 Treatment by Spray Drying

Several factors explain why spray‐dried egg whites have international importance. Due to the antimicrobial proteins (Table 1) in egg white, raw egg whites do not promote bacterial growth like egg yolk, allowing large quantities to be transported to specialized drying facilities. Another important point is that egg whites lose weight when the water is removed, which leads to lower transportation costs. Egg whites can be stored for longer periods without spoiling because they are fat‐free. To prevent browning caused by the Maillard reaction during drying, carbohydrates are removed from the egg white before further processing. Glucose is removed through fermentation with starter cultures, such as yeasts or LAB (Table 2) (Asaithambi et al. 2022). Alternative methods include enzymatic removal of glucose using glucose oxidase or chemical removal using H_2_O_2_. After glucose is removed, the egg whites are dried using spray drying or pan drying. Spray drying is the standard industrial process. To reduce residence time in the spray tower and lower drying costs, the water content of the egg whites is often reduced through reverse osmosis before drying (Grumezescu and Holban 2018; Asaithambi et al. 2022).

A peculiarity of the egg white drying process is that pasteurization after spray drying occurs as long‐term pasteurization. Pasteurization is then carried out by heating the powder in a chamber with specific time‐temperature parameters, typically at 65°C for at least 10 days. This treatment ensures the microbiological safety of the product (Wei et al. 2020). However, more intense heat treatments can be used, such as applying 80°C for 5–10 days during chamber drying or about 90°C for 20 h in dynamic drying with a conical agitated mixer. These higher temperatures not only destroy bacteria but also affect foaming stability (Gharbi and Labbafi 2019; Wei et al. 2020). To address this issue, in Europe, triethyl citrate (E1505) is added to egg white to enhance foam stability after reconstitution (European Commission 2011).

On the other hand, the emulsifying and/or gelling properties of the protein are improved during the spray drying process. The improvement of these functional properties is the result of protein modifications, including the increase of surface hydrophobicity, molecular flexibility, and the exposure of reactive residues (Wei et al. 2020; Katekhong et al. 2018; Hu et al. 2025; Jin et al. 2022).

Nowadays, to reduce transportation costs and extend shelf life, most companies produce egg white powder with specifically modified functional properties to offer targeted products for particular applications, such as high‐gel egg white powder for confectionery, bakery, and dessert products (Ma et al. 2021; Razi et al. 2023; Wang et al. 2023).

For food safety, pasteurizing egg whites is essential, but functional properties also play an important role for manufacturing (Bermudez‐Aguirre and Niemira 2023; Zhu et al. 2018; Wang et al. 2019; Jun et al. 2020; Tian et al. 2024). Bhat et al. (2021) and Liu et al. (2019) provide a detailed overview of how processing affects the digestibility and functional properties of egg white products.

However, the thermal sensitivity of proteins and the associated instability within the effective pasteurization temperature range of liquid egg white pose a challenge during processing (Jiang et al. 2024). Heat‐resistant microorganisms can survive or adapt at temperatures too low for pasteurization, spoil the product, or cause foodborne illness even when refrigerated (Necidová et al. 2019).

### 5.4 Novel Pasteurization Techniques of Egg White

#### 5.4.1 Novel and Emerging Thermal Technologies

Manufacturers expect safe and optimized products for new applications; therefore, an increase in research into new methods that can selectively modify the functionality of egg proteins while ensuring a safe and microbiologically stable egg white product can be recorded. More attention is being directed toward new nonthermal techniques, such as PEFs, HPP, pulsed light (PL), microwave (MW), ultraviolet light (UV), ultrasound (US), cold plasma (CP), and ohmic heating (OH). These methods can inhibit microbial growth and, in some cases, even replace homogenization steps, thereby reducing the number of process steps (Al‐Najjar et al. 2023).

#### 5.4.2 Pulsed Electric Field

The permeability of the cell membranes of vegetative microorganisms in liquid media increases (via electroporation) during the PEF technique through the application of short, high‐voltage pulses (Yogesh 2016; Zhang et al. 2021). This process causes the cell membrane to lose its natural barrier function and activity. The membranes of PEF‐treated cells become permeable to small molecules (Yogesh 2016); this permeation leads to swelling of the cell membrane and eventual rupture or loss of cell organelles through pores (Yogesh 2016).

During processing, the product is exposed to short, high‐intensity electric fields (approximately 20–80 kV). Increasing the temperature of the egg white to 30–40°C before treatment can improve pasteurization results. Some studies have shown the potential for egg white processing with PEF (Baba et al. 2018). The main advantage over conventional preservation methods is the lower temperature stress. Wu et al. (2016) demonstrated that soluble protein content, which correlates with functional properties, decreased by 8.78% during PEF treatment (25 kV/m; 800 µs) and by 23.95% during thermal treatment (61°C; 4 min). Therefore, PEF treatment has shown to enhance gelling ability and viscosity, opening new possibilities for use in the food industry (Yogesh 2016; Gharbi and Labbafi 2018). In the study by Shams et al. (2024), the effects of PEF on functional properties are discussed in more detail.

#### 5.4.3 High Hydrostatic Pressure

Another effective method of inactivating microorganisms is HPP (Pou and Raghavan 2020; Gharbi and Labbafi 2018). HPP involves applying pressures ranging from 200 to 700 MPa for a few minutes. The effect of HPP on eggs was first studied by Bridgman (1964), who observed coagulation of the egg gel at pressurization of 600 MPa. Studies have already shown that *L. monocytogenes*, *Salmonella* spp., and *E. coli* can be successfully killed by this method. HPP reduced *S. enteritidis* when applied to liquid whole eggs at 350 MPa and 50°C for 2 min pulses over four cycles. Its potential for processing egg products has been extensively studied (Pou and Raghavan 2020; Tóth et al. 2020; Yang, Rao et al. 2021; Gharbi and Labbafi 2018; Woldemariam and Emire 2019). Coagulation of the egg can be prevented by adding 7%–10% sodium chloride or sucrose, even at pressures of 800 MPa (Liu et al. 2019).

Other changes in egg components have been reported, such as the enhancement of the foaming ability of egg whites due to exposure of SH groups, which promotes foaming stability (Naderi et al. 2017). Increasing the foaming ability and stability of egg whites can be beneficial in food manufacturing, especially in producing pastries and desserts. A comprehensive overview of the influence of HPP on the functionality and nutritional properties of proteins and egg products is provided by Wang, Yang, et al. (2022) and Naderi et al. (2017).

HPP is generally described as a technology that can increase the activity of some enzymes (Tribst et al. 2017). Tribst et al. (2017) showed in their study that muramidase activity and antimicrobial properties in egg white improved after treatment with HPP. This not only enables mild pasteurization but also could improve storage stability.

Until now, there has been no regulation for the HPP treatment of egg whites. Most products in the EU that undergo HPP treatment are classified as novel foods if the HPP process changes the composition or nutritional value. They are, therefore, subject to the relevant Novel Food Regulations. This needs to be assessed on a case‐by‐case basis. Food manufacturers are obligated to verify whether a food or food ingredient falls under the Novel Food Regulation (Aganovic et al. 2021).

Countries outside the EU, such as the United States, Australia, and New Zealand, require that a minimum reduction of relevant microorganisms of 5 logs (logarithmic) be consistently achieved through HPP treatment of egg white (Aganovic et al. 2021; Stewart et al. 2016).

#### 5.4.4 MW Pasteurization

Another method for pasteurizing egg whites is MW pasteurization. MW pasteurization offers several advantages over conventional methods, such as faster and more energy‐efficient treatment (Liu et al. 2022). The benefit of MW pasteurization is the quick and even heating of the egg white by MW energy, which allows for continuous processing and reduces treatment time compared to traditional methods (Yang, Sun, et al. 2023; Wang, Gu, et al. 2020b). A study by Yang, Sun, et al. (2023) examined the effects of MW pasteurization on the microbial load of egg whites. The authors found that MW pasteurization effectively kills harmful microorganisms such as *Salmonella* spp. (−4.945 ± 0.066 log_10_ at 60°C) and *E. coli* (−5.050 ± 0.145 log_10_ at 60°C) while improving the functional properties of the egg white. Additionally, the foaming properties could be improved between 5.6% and 7.95% compared to untreated samples.

#### 5.4.5 UV Light Treatment

UV light treatment of egg white is a nonthermal method for pasteurization. The egg white is exposed to UV light to eliminate microorganisms. UV radiation chemically interacts with DNA and other cell components. As a result, damaged cells can no longer reproduce. Light in the 250–260 nm range is especially effective at inactivating microorganisms because their DNA absorbs radiation in this spectrum. How deeply UV radiation can penetrate limits the sterilization of liquid foods. In highly turbid foods, radiation only reaches a thin surface layer. That is why industry often uses thin liquid films (thin liquid reactors) or mixed reactors (spiral or turbulence reactors). Some studies in the literature have explored UV treatment of egg whites (Gharbi and Labbafi 2018).

A study by Ouyang et al. (2019) investigated the effects of UV treatment of egg whites. The results revealed that UV treatment (7 mL, 40 s, distance 5 cm) could effectively reduce the number of *E. coli* (1.01 log CFU/mL and *S. enteritidis* (1.73 log CFU/mL). The study showed that pulsed UV light does not change the foaming ability (4.4 cm) or foam stability (19 mL) of liquid egg white compared to untreated sample (4.5 cm; 17.6 mL). However, UV treatment of egg white has some potential disadvantages. Irradiation with UV light can lead to changes in protein structures and affect functional properties (Gharbi and Labbafi 2019). Currently, there are no known studies addressing changes in the taste of egg whites due to UV irradiation.

#### 5.4.6 Ultrasonic Pasteurization

Ultrasonic pasteurization (US) provides an alternative to conventional heat treatment by offering an effective reduction of microorganisms. The treatment of proteins with US is a promising method to influence their structure and function (Chen, Sheng, et al. 2019b; Sheng et al. 2018; Stefanović et al. 2018). US consists of sound waves with a frequency above the human hearing threshold. It can affect proteins in several ways, including denaturation, enzyme activation, and modification of protein structures (Chen, Sheng, et al. 2019b; Liu et al. 2022; Sheng et al. 2018; Stefanović et al. 2018). Some studies have investigated the use of US to pasteurize egg whites. The authors showed that US treatment can reduce harmful microorganisms, such as *E. coli*, while largely preserving the functional properties of egg whites, such as gel‐forming ability and emulsion properties (Liu et al. 2022; Sheng et al. 2018; Stefanović et al. 2018).

A study by Chen, Sheng, et al. (2019a) examined the effects of ultrasound on the foaming and physicochemical properties of egg whites. The results revealed that ultrasound can enhance the foaming ability (80%–100%) of treated egg whites compared to untreated (30%–50%) (Chen, Sheng, et al. 2019a). US pasteurization offers several advantages over traditional methods. It is more energy‐efficient because it operates at lower temperatures. Additionally, it allows continuous processing of protein. Moreover, US pasteurization can inactivate enzymes, which help extend shelf life. Overall, research indicates that US pasteurization is a promising method to safely and gently sanitize protein while preserving its quality and nutrients. However, further studies are necessary to identify the optimal US treatment duration and intensity for different protein products (Liu et al. 2022; Agregán et al. 2023). Li et al. (2023) provide a comprehensive review of the impact of ultrasound and MWs on food.

#### 5.4.7 Cold Plasma

CP sterilization is a promising method for disinfecting egg whites. CP refers to an ionized gas state generated at room temperature. It produces various reactive species, such as free radicals and UV radiation, which can inactivate microorganisms (Bermudez‐Aguirre and Niemira 2022; Agregán et al. 2023; Liao et al. 2019). CP sterilization offers the advantage of rapid treatment with a 1.94 log reduction for *E. coli* and 1.11 log reduction for *S. enterica* in 60 s (Movasaghi et al. 2025). This enables continuous processing of egg whites and reduces treatment time compared to conventional sterilization methods. However, this method has not yet been approved for the treatment of egg whites (Zhang et al. 2022).

Treating egg whites with new technologies offers promising opportunities to enhance the functional properties of this important food ingredient and broaden its applications in the food industry. Alternatives such as HPP, PEF, or combined methods (e.g., mild heating and US) show promising results in improving safety and functionality. In our view, more emphasis should be placed on combined, mild procedures in future applications to preserve the natural bioactive and functional properties of egg white. Over the long term, using innovative, nonthermal processes alongside smart packaging and cold chain monitoring seems promising.

#### 5.4.8 Ohmic Heating

OH is an innovative thermal process where electrical energy is directly converted into heat within a conductive food product. An electric current flows through the product, heating it quickly and evenly. Because of its high moisture content and dissolved ions, egg white has enough electrical conductivity to be effectively heated using OH (Alkanan et al. 2021). Studies indicate that OH provides a more uniform temperature distribution than traditional heating methods and can reduce thermal damage (Balakrishnan et al. 2024). Additionally, OH can inactivate microorganisms (5 log at 60°C in 2.91 min) and enzymes while maintaining protein functionality (e.g. water holding capacity (wt%) OH 15.70 ± 3.23 vs. conventional heating 10.51 ± 1.11), making it a promising egg white pasteurization method (Joeres et al. 2022; Balakrishnan et al. 2024; Pires et al. 2021).

Many egg white pasteurization studies are done in labs, which do not take the effect of the matrix into account. Differences in systems and lack of standards make comparison difficult (Table 4).

Functional properties, such as foaming capacity, gelation, and solubility, are often insufficiently considered, even though they are of central importance for application in food processing. A limitation in the current body of research is the use of different types of raw material, such as fresh, frozen, or pasteurized egg white, which can vary in composition and initial microbial load. Additionally, diverse methodological approaches, including differences in time‐temperature regimes, equipment, and process parameters, make it difficult for direct comparisons or establish generalizable conclusions.

## 6 Biotechnology of Egg White

### 6.1 Biotechnological Applications

Biotechnological applications of hen egg white proteins have become increasingly significant in food technology, as well as in pharmaceutical and biomedical research. Because of their antimicrobial and functional properties, they are used as natural additives in food processing, to enhance texture or for microbial stabilization. Lysozyme is already used commercially as a preservative in cheese or wine. In biomedicine, egg white proteins serve as suitable starting materials for developing functional peptides or active pharmaceutical ingredients due to their bioactivity (e.g., antimicrobial and antioxidant) (Abeyrathne et al. 2018; Liu et al. 2018). Egg white peptides can promote wound healing and have the potential to accelerate healing at the cellular level in cases of mechanical skin damage. Functional peptides from egg white represent a new therapeutic option for skin treatment and wound healing (Ge, Jiang, et al. 2021). Egg white's high availability, diversity, and biological activity make it a valuable biotech resource. In this review, only selected aspects of food technology will be discussed. The pharmaceutical applications and other uses will be examined deeper in the review by Liu et al. (2024).

### 6.2 Fermentation of Egg White

#### 6.2.1 Lactic Acid Bacteria in Egg White Fermentation

Besides chemical and physical processes, fermentation is a specific method of altering food properties and extending shelf life, including egg products (Prado Barragán et al. 2016; Thierry et al. 2023). Microbial cultures or their enzymes are involved in this transformation, affecting the food's sensory and nutritional qualities (Jiang et al. 2020; Mangalisu et al. 2022).

Unlike other fermented foods, there has been little research on using egg white as a raw material. Microbial cultures are already used in producing egg products, for example, to prevent browning caused by the Maillard reaction during drying (Table 2) (Delves‐Broughton 2014).

**FIGURE 6:**
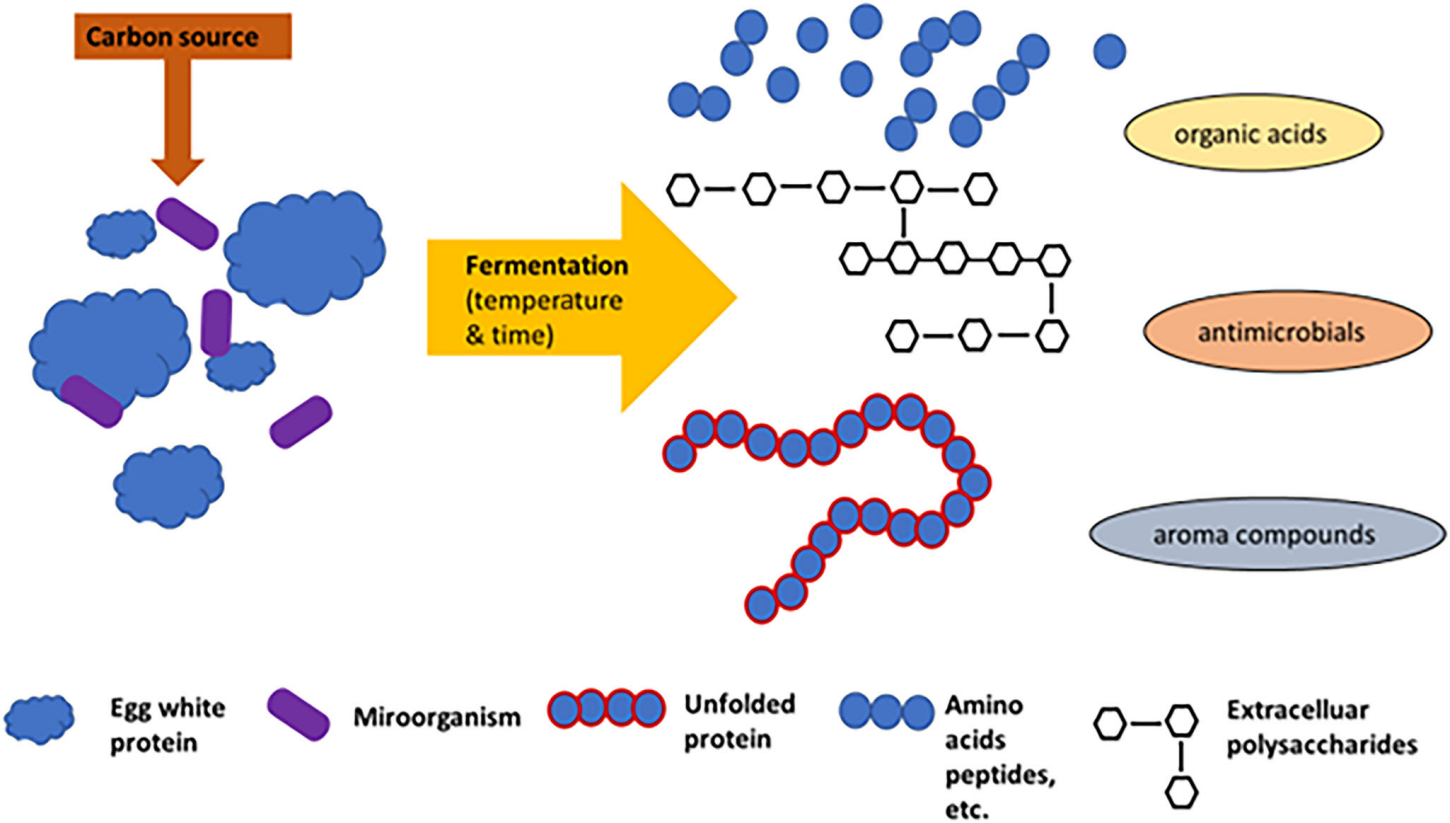
Schematic illustration of the fermentation of egg white by selected microorganisms. Microorganisms, for example, LAB, modify egg white by utilizing it and carbon sources to produce substances such as organic acids, extracellular polysaccharides, antimicrobials, or aroma compounds. During fermentation, proteases are released that hydrolyze proteins (own representation, based on Mourad et al. 2023; Chen, Wang et al. 2022; Lv et al. 2024; Jia et al. 2022; Wang, Xu, et al. 2022; Chen, Han et al. 2022; Jiang et al. 2020).

In recent years, fermenting egg white with LAB has become more significant in the literature due to improved characteristics and the development of new products (Chen, Wang, et al. 2022; Jia et al. 2022; Wang, Xu, et al. 2022). Egg white fermentation involves enzymatic and microbial changes to proteins and carbohydrates, leading to the creation of bioactive compounds and metabolites (Figure 6) (Mourad et al. 2023; Chen et al. 2024; Lv et al. 2024).

Furthermore, the biopreservative properties of Lactobacilli enable egg white to be preserved without the need for chemical preservatives. Additionally, LAB provides a health‐promoting benefit that can positively impact the health of consumers (Jiang et al. 2020; Mangalisu et al. 2022). Different strains of LAB suitable for the fermentation of egg white are shown in Table 3.

**TABLE 3:** Influence of pasteurization and preservation processes on egg white: microbiological reduction and functional properties.

Pasteurization process	Condition	Microbial reduction	Influence on functional properties	References
Conventional heat pasteurization	55–68°C for 3–6 min	≥5 log (*Salmonella* sp.)	Significant loss of foam formation (up to 72%); slight improvement in foam stability; protein aggregation observed	Fan et al. (2024), Liu et al. (2022)
Spray drying	150–180°C inlet, short residence time	ni	Denaturation of proteins reduces functionality (e.g., solubility, foaming); often, reduced rehydration	Katekhong and Charonrein (2017)
Refrigation	0–4°C	No reduction; growth slower	Functional properties decrease; suitable for short‐term preservation	Wang, Liu, et al. (2020)
Freezing	−18°C	No reduction; growth slower	Foaming ability decreased	Dai et al. (2022)
UV irradiation	Distance 5 cm, 40 s, 45.6 J/cm^2^	1.28 log CFU/mL (*Escherichia coli* K12), 1.98 log CFU/mL (*Salmonella enteritidis*)	No change in foaming ability or foam stability	Ouyang et al. (2019)
High‐pressure‐processing (HPP)	350–500 MPa, 5–15 min, 20–25°C	≥5–7 log (*Salmonella* sp.)	Foam density increased; foam stability increased; a creamy appearance	Singh and Ramaswamy (2015)
Microwave (MW)	55–60°C, <1 min	≥5 log (*E. coli*)	Improved foam formation	Liu et al. (2022)
Ultrasound (US)	700 W, 4.5 min	<4 log CFU/mL (*E. coli*)		Liu et al. (2022)
	60 Hz, 400 W, 15 min	ni	Foam stability increased	Zhang et al. (2024)
Ohmic heating (OH)	68°C for 5 min	≥5 log (*Salmonella* sp.)	Improved foam formation and stability; reduced protein aggregation compared to conventional heat	Balakrishnan et al. (2024)
Cold plasma (CP)	2.2 and 4.2 kV for 0–40 min under atmospheric conditions	3–5 log	Improved foam formation; decreased stability; improved emulsion stability (until 30 min treatment)	Baek et al. (2021)

Abbreviation: UV, ultraviolet light.

**TABLE 4:** Overview of starter cultures used in egg white processing—strains, matrix, and potential effects.

Microorganisms	Matrix	Influenced characteristic	References
*Saccharomyces cerevisiae*	Egg	Desugarization of egg products	Sharma et al. (2012)
*Streptococcus thermophiles*, *Lactobacillus bulgaricus*	Egg white	Rheological and foaming activities	Jiang et al. (2020)
*Aspergillus oryzae*	Egg white	IgE binding capacity was reduced	Li et al. (2018)
*Lactobacillus delbrueckii* spp. *bulgaricus, Lactobacillus acidophilus, Streptococcus thermophilus, Bifidobacterium animalis*	Egg white	Foaming properties	Jia et al. (2021)
*Lactobacillus bulgaricus, Lactobacillus acidophilus, Streptococcus thermophilus*	Egg white with full cream milk powder	Influence of milk powder and fermentation time on the microbial count and pH decrease	Nahariah et al. (2019)
*Lactobacillus plantarum*	Fresh liquid egg white	Dried egg white	Nahariah et al. (2018)
*Lactiplantibacillus plantarum*	Lysozyme‐free egg white	Gel properties of lysozyme‐free egg white before and after fermentation	Chen, Wang, et al. (2022)
*Lactobacillus bulgaricus, Lactobacillus acidophilus*	Egg white, milk powder	Change of aroma, taste, preference of consumers, and color with increasing fermentation time	Milawati et al. (2020)
*Lactobacillus bulgaricus*	Egg white powder	Improved functionality	Azizah et al. (2022)
LAB	Egg white	Egg white yoghurt, pH‐induced gelling	Zang et al. (2023)
LAB	Egg white	Lactic‐fermented egg white improves visceral fat obesity in Japanese subjects	Matsuoka et al. (2017)
LAB	Egg milk	Beverage	Lyu, Yang, et al. (2023)
*Lactobacillus acidophilus, Lactobacillus brevis, Lactobacillus bulgaricus, Lactobacillus casei, Lactococcus lactis, Pediococcus cerevisiae, Pediococcus pentosaceus, Leuconostoc mesenteroides, Streptococcus thermophilus, Bifidobacterium longum*	Liquid egg white	Yoghurt	Young et al. (2019)
*Lactobacillus bulgaricus, Streptococcus thermophilus*	Fresh liquid egg white	High protein egg white yoghurt	Li et al. (2023)
*Lactobacillus delbruckii* subsp. *bulgaricus, Streptococcus thermophilus*		Fermented egg product	Asunmaa and Mäkelä (2023)
*Streptococcus thermophilus, Lactobacillus bugaricus*	Liquid egg white	Fat‐free, cholesterol‐free fermented drink	Wu and Zheng (2019)
LAB	Liquid egg white	Egg white biological beverage based	Li (2018)
*Streptococcus thermophilus, Lactobacillus bulgaricus*	Egg white powder	Egg white peptide puddings with probiotics	Yao and Dongrong (2020)
*Lactobacillus plantarum, Lactobacillus bulgaricus, Streptococcus thermophilus*	Liquid egg white	Low‐fat fermented protein dried	Yang, Chen et al. (2021)
LAB	Native egg white	Screening for suitable LAB	Lv et al. (2024)
*Lactobacillus plantarum, Pediococcus pentasaceus*	Egg white	Reducing the off‐flavor of egg white powder	Chen et al. (2024)
*Lactobacillus casei*	Egg white beverage	Shelf life of fermented egg white based beverage	Mourad et al. (2023)
*Streptococcus thermophilus*	Egg white	Enhancing the rehydration capacity and foaming property of spray‐dried egg white	Jia et al. (2024)
*Streptococcus thermophilus*	Egg white and milk	Flavour enhancement	Gao et al. (2025)
*Streptococcus thermophilus*	Native egg white	Influence of gelling properties	Chang et al. (2024)

Abbreviation: LAB, lactic acid bacteria.

#### 6.2.2 Influence of Egg White on Lactic Acid Bacteria

Although antimicrobial proteins like avidin, lysozyme, and ovotransferrin are naturally present in egg white, several studies have demonstrated that fermenting egg white with microorganisms is possible (Jiang et al. 2020; Zang et al. 2023). Most studies and patents show that egg white is pasteurized at below 90°C before fermentation, which could suggest that these antimicrobial proteins avidin (70–85°C), ovotransferrin (61–65°C), protease inhibitor (77°C), and lysozyme (69–77°C) are damaged during pasteurization (Table 1) (Baron et al. 2016; Jabalera et al. 2022; Liao et al. 2018; Stefanova et al. 2021). However, the study by Wang, Xu, et al. (2022) indicates that native proteins can be fermented, implying that protein denaturation is not necessary for fermentability with LAB. This is confirmed in the study by Carrillo et al. (2014), who showed that lysozyme does not affect *Lentilactobacillus hilgardii*, *L. plantarum*, and *Lactobacillus casei*.

Instead, the pH of the natural egg matrix seems to affect microbial growth. As discussed earlier, the pH of fresh, unprocessed egg white usually ranges from 7.6 to 9.7, depending on storage conditions and duration (Dabestani and Yeganehzad 2019). Several studies have emphasized the impact of this alkaline pH on proteins and antimicrobial activity (Alabdeh et al. 2011). These studies demonstrated that, compared to pH 7.5 or 8, egg white shows greater antimicrobial activity at pH levels above 8.8.

Ovotransferrin is unlikely to affect LAB (Legros et al. 2021) because it primarily inhibits Gram‐negative bacteria. Egg white contains a high level of active protease inhibitors (ovomucoid, ovoinhibitor, ovostatin, and cystatin). Proteases are enzymes that break down proteins and are necessary for many biological processes, including breaking down peptide chains. The presence of protease inhibitors in eggs inhibits bacteria that produce proteases (Guyot et al. 2016). Furthermore, different strains of *L. rhamnosus*, *L. paracasei*, and *L. plantarum* have shown strain‐specific resistance to lysozyme in various studies (Vilcacundo et al. 2018). This resistance is due to the *O*‐acetylation of the *N*‐acetyl‐muramic acid in peptidoglycan (Chapot‐Chartier and Kulakauskas 2014; Yadav et al. 2018).

Studies by Chen, Wang, et al. (2022), Gundogan et al. (2023), and Jia, Ji et al. (2021) demonstrated that fermentation of egg white is possible with *Lactobacillus acidopillus*, *Lactobacillus delbrueckii*, *L. plantarum*, *L. rhamnosus*, and *Lactobacillus sanfrancisensis*. *Lactobacillus* spp., *Streptococcus diacetilactis*, *Klebsiella pneumoniae*, and *Saccharomyces cerevisiae* have long been used in the egg industry to remove carbohydrates from egg white before drying.

#### 6.2.3 Influence of Fermentation on Egg White

Chen, Wang, et al. (2022) investigated the effects of fermentation on the physical and chemical properties of egg whites. The results showed improved structure, increased viscosity, and enhanced antioxidant activity after fermentation. Fermentation of egg whites with LAB can influence the taste and flavor. A study by Milawati et al. (2020) examines the influence of fermentation on the taste of egg white. The results showed an increased umami note and improved flavor intensity after fermentation. Fermentation can be an effective tool to modify the aroma of egg whites and reduce their characteristic off‐flavors. With lactic acid fermentation, it is possible to alter the volatile profile by increasing the formation of aldehydes, ketones, alcohols, esters, and organic acids (Sun et al. 2025; Chen et al. 2024; Gao et al. 2025; Jia et al. 2025).

In particular, fermentation, when combined with enzymatic hydrolysis, has been shown to effectively reduce undesirable off‐flavors in egg whites, such as those derived from lipid oxidation. At the same time, the formation of positive sensory compounds, including esters, is promoted. Studies focusing on applications further indicate that using fermented egg white ingredients can improve the aroma quality and overall sensory acceptance of formulated food products (Chen et al. 2024; Gao et al. 2025; Jia et al. 2022, 2025).

Overall, the available literature shows fermentation as a versatile tool for targeted aroma modification of egg white proteins. However, the extent and direction of aroma development depend heavily on the selected microbial cultures, processing conditions, and potential enzymatic pretreatments. This shows the need for controlled processes in future industrial applications (Chen et al. 2024; Gao et al. 2025).

In addition to flavor formation, various LAB have antimicrobial activity and improve the shelf life of food (Prabhurajeshwar and Chandrakanth 2019).

Further research is necessary to determine whether fermentation can enhance the bioavailability of pasteurized egg whites. It is unclear if the interaction between egg white components and LAB contributes to microbial stability or if certain strains merely tolerate the antimicrobial environment. More research is needed to clarify the mechanisms behind LAB survival and functionality in egg white matrices. Chen, Wang, et al. (2022) demonstrated the effects of fermentation with *L. plantarum* on the gelation properties of lysozyme‐free egg whites. Removing lysozyme from egg white reduces its gelling ability. More desirable gelation is observed following fermentation (Wang, Xu, et al. 2022).

Eggs are high in protein and vitamins, but they can cause allergies. They are among the most commonly reported food allergens, often affecting children. The main allergens are ovalbumin, ovomucoid, ovotransferrin, and lysozyme from egg white. Some studies show that fermentation with LAB can reduce egg white allergens (Dhanapala et al. 2015; Ma et al. 2020; Zhu et al. 2018; Li et al. 2018). Table 3 summarizes the microorganisms used. Fermented egg products could expand the egg product market by offering new sensory experiences. Specifically, by using side streams of the egg industry, manufacturers can create value‐added and sustainable products.

### 6.3 Functional Peptides From Egg White

#### 6.3.1 Manufacturing of Egg White Peptides

Eggs are a source of bioactive compounds, including functional peptides. With more than 50 amino acids, they are classified as proteins (Zhu et al. 2018). These peptides are derived from various egg components such as egg white, egg yolk, and eggshell membranes and possess a range of bioactive properties that make them appealing for research and development in the fields of nutrition, medicine, and cosmetics (Jain and Anal 2017; López‐Martínez et al. 2021; Zhao et al. 2019).

These peptides containing essential amino acids are easily absorbed sources of high‐quality protein necessary for muscle recovery, immune function, and overall health (Jia et al. 2021). Peptides derived from egg white can form in the intestine during protein digestion. The finding that these peptides have a biological effect on the body is relatively recent. This effect is based on the ability of peptides to be taken up by special peptide transporters in the gut, circulate in the blood, and interact with cells and tissues throughout the body (Bhat et al. 2021; Wang, Qiu, et al. 2018; Puglisi and Fernandes 2022).

**FIGURE 7:**
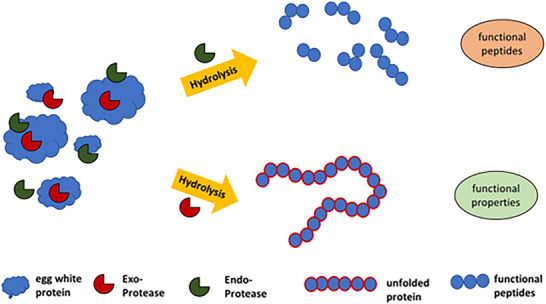
Mechanism of action for the production of egg white protein hydrolysates (based on Lyu, Chen, et al. 2023; Carrillo et al. 2018; Ho et al. 2021) (own representation, based on Wang, Gu, et al. 2020a; Grootaert et al. 2017; López‐Martínez et al. 2021; Loveday 2023; Nasri 2017; Zhang et al. 2020; Jain and Anal 2017; Chen et al. 2024).

Functional peptides can be derived from egg white through hydrolysis. Hydrolysates can be generated either by acid treatment under pressure or through enzymatic hydrolysis (Garcés‐Rimón et al. 2016; Ling et al. 2020; López‐Martínez et al. 2021). These bioactive peptides can be produced via in vitro enzymatic hydrolysis or microbial fermentation. Enzymatic hydrolysis is the most commonly used method (Jain and Anal 2017; Chen et al. 2024), involving the addition of proteases (e.g., pepsin, trypsin, chymotrypsin, papain, and alcalase) to the raw material. As shown in Figure 7, during fermentation, proteins are broken down by proteases released into the medium by microorganism (e.g., LAB) (Bhat et al. 2021). Table 5 lists specific proteolytic enzymes used in food technology for producing egg white protein hydrolysates, along with their classification into endopeptidic and exopeptidic proteases and their mechanisms of action. Endopeptidases, such as trypsin, pepsin, or alcalase, mainly cleave peptide bonds within the protein structure at specific amino acid residues, producing larger peptide fragments. Exopeptidases, such as aminopeptidases or carboxypeptidases, catalyze the removal of individual amino acids from the N‐ or C‐terminus. The choice and combination of these enzymes affect the degree of hydrolysis, peptide profile, and the functional and sensory properties of the hydrolysates (Wang, Gu, et al. 2020a; Grootaert et al. 2017; López‐Martínez et al. 2021; Loveday 2023; Nasri 2017; Zhang et al. 2020).

**TABLE 5:** Commonly used proteases for the production of egg white protein hydrolysates, enzyme, raw material, and the enzymatic effect.

Enzym	Raw material	Enzymatic effect	References
Protease	Native and heated egg white	Antibacterial peptides	Carrillo et al. (2018)
Neutral protease	Egg white	Antioxidant activity	Kobayashi et al. (2017)
Chymotrypsin and pepsin	Liquid egg white	Antioxidant activity	Yuan et al. (2020)
Alcalase	Egg white protein	Antioxidant activity	Noh and Suh (2015)
Proteinase K	Egg white protein	Antioxidant activity	Brumă et al. (2024)
Alcaline protease	Egg white	Anti‐inflammatory and antioxidant	Ge, Cai, et al. (2021)
Neutral protease	Egg white powder	Improved foaming	Lyu, Chen, et al. (2023)
Phospholipase A	Egg white	Improved foaming and storage ability	Yüceer and Caner (2022)
Phospholipase A	Liquid egg white	Improved foaming	Yüceer (2020)
Protease A	Native egg white	Improved foaming	Ho et al. (2021)
Flavourzyme, *Lactobacillus plantarum* and *Pediococcus pentosaceus*	Native egg white	Improved flavour	Chen et al. (2024)
Alkaline protease, neutral protease, LAB	Native egg white	Improved flavour	Gao et al. (2025)

Abbreviation: LAB, lactic acid bacteria.

Nevertheless, it is important to understand the differences between these two mechanisms and their practical application. Enzymatic hydrolysis is a process that offers a high degree of process control and reproduceability. Furthermore, it results in shorter processing times and well‐defined peptide profiles, depending on the enzyme (Song et al. 2024). In contrast, microbial fermentation has shown to generate a more extensive range of peptides through the action of multiple proteases. However, this process often leads to less predictable peptide compositions and longer processing times. Although fermentation may offer additional functional or sensory benefits, enzymatic hydrolysis is currently regarded as a more suitable process for industrial‐scale production due to its controllability and scalability (Gao et al. 2025; Chen et al. 2024; Song et al. 2024; Jain et al. 2017).

A comprehensive overview of the regulatory and safety rules for bioactive peptides is provided by Patil et al. (2022).

#### 6.3.2 Improved Functional Properties

The food industry is increasingly focused on enzymatic protein hydrolysates and various purified peptides. Numerous studies have been conducted on the enzymatic hydrolysis of egg white proteins and the characteristics of the products (Wang, Gu, et al. 2020a; Grootaert et al. 2017; López‐Martínez et al. 2021; Loveday 2023; Nasri 2017; Zhang et al. 2020). These studies examined enhanced functionalities, such as solubility, emulsification, foaming properties, and aroma qualities (Ho et al. 2021; Liu et al. 2018, 2019; Loveday 2023; Nasri 2017).

Ho et al. (2021) showed that pasteurization reduced egg white foamability, decreasing overrun from 621.3% ± 0.10% to 575.5% ± 0.16%, whereas the addition of egg white hydrolysates to pasteurized egg white increased overrun to 625.3% ± 0.15%. According to López‐Martínez et al. (2021), egg white hydrolysate is suitable for replacing dairy in ice cream. Furthermore, this new nondairy functional ice cream has very low sugar and fat content and similar sensory properties to commercial dairy ice cream (López‐Martínez et al. 2021).

#### 6.3.3 Antioxidative Activity

Besides improved functional properties, antimicrobial and antioxidant activities have been demonstrated. In their study, Zheng et al. (2020) showed that egg white peptides have antioxidant effects and upregulate antioxidant enzymes in the body, thereby protecting cells from oxidative stress. Six peptides were identified as having antioxidant effects based on reduced superoxide formation and increased levels of superoxide dismutase and catalase in cells. This study provided evidence for naturally occurring antioxidant peptides in egg whites. The antioxidant peptide Asp‐His‐Thr‐Lys‐Glu in egg white contains specific amino acids associated with zinc supply (Wang, Gu, et al. 2020a; Zhang et al. 2020; Nimalaratne et al. 2015; Chen, Han, et al. 2022).

#### 6.3.4 Antibacterial Activity

Egg white peptides have antibacterial properties and can inactivate various bacterial strains, including *Bacillus* spp., *Staphylococcus* spp., *Listeria* spp., and *Salmonella* spp. They can interact electrostatically and hydrophobically with the cell wall and cell membrane of susceptible bacteria, resulting in increased cell permeability and ultimately leading to lysis of the cell, which causes the death of the bacteria (Carrillo et al. 2018; Moreno‐Fernández et al. 2020; Liao et al. 2018). In the study by Carrillo et al. (2018), native chicken lysozyme hydrolysates exhibited antibacterial activity, leading to a reduction of 5.3 log in *E. coli* and 6.8 log in *Staphylococcus carnosus*. In contrast, heat‐treated hydrolysates showed lower antimicrobial activity, with reductions of 0.8 and 1.0 log for *E. coli* at pH 6.0 and 7.0, respectively, and 2.2 and 2.3 log for *S. carnosus* under the same conditions.

#### 6.3.5 Health Benefits

Functional peptides are useful beyond the food industry. According to several studies, the peptides in egg white can help slow inflammation in the intestines. They also can help relieve colitis symptoms and intestinal damage by providing anti‐inflammatory effects, restoring the intestinal mucosa, and modulating the gut microbiota (Yang, Lyu, et al. 2023; Zhou et al. 2022).

Some peptides in egg white can act as potent angiotensin‐converting enzyme inhibitors, which help lower blood pressure. These peptides can be bifunctional, serving as antioxidants and having fewer side effects than commercially available drugs and exerting multiple biological effects (Ding et al. 2016; Grootaert et al. 2017; Zhang et al. 2020).

The peptides seem to enhance insulin release after eating and reduce appetite through glucagon‐like peptide‐1. Therefore, discovering the peptide sequences and receptors involved in hormone release might lead to new ways to manage food intake and glucose metabolism in humans (Santos‐Hernández et al. 2020).

Egg‐derived functional peptides are promising in nutrition, medicine, and cosmetics because of their range of biological activities, abundant availability, and high biocompatibility. Further research into the molecular mechanisms and therapeutic uses of these peptides could unlock all their potential benefits.

## 7 Conclusion

The review shows the complex interplay between safety, functionality, and processing technologies in egg white products. Pasteurization and hygienic processing have improved microbiological control, but challenges remain, with psychrotrophic and heat‐resistant micro organisms like *Pseudomonas* spp. and *Bacillus* spp., which can adapt to egg white's antimicrobial environment. Although antimicrobial components and the naturally high pH of fresh egg white offer intrinsic protection, their effectiveness is limited under certain conditions.

Technological parameters, including pH, temperature, and storage duration, play a role in determining both microbial and functional activity, such as foaming and gelling properties. This dual impact underlines the need for carefully balanced processing strategies.

Biotechnological approaches, especially fermentation, offer promising alternatives. They not only enhance microbial safety through the formation of organic acids and antimicrobial peptides but also enable targeted modification of functional properties aligning with clean label and sustainability goals. Combining mild heat treatments with controlled fermentation and “hybrid strategies” appears promising in microbial risks and functional requirements.

There's a need for standardized studies using consistent starting materials and real‐world scenarios to better understand microbial dynamics in egg white. Special attention should be given to viral and mycotoxin contamination, topics that remain under‐researched despite potential relevance to food safety. The safe and functional processing of egg white requires an approach integrating microbiology, technology, and product development. With further interdisciplinary research, egg white has the potential to evolve into a next‐generation functional ingredient that meets industrial and consumer expectations.

## Author Contributions

**Insa Mannott**: writing – original draft, conceptualization, project administration. **Ramona Bosse**: conceptualization, writing – review and editing, funding acquisition, supervision. **Monika Gibis**: conceptualization, writing – review and editing, supervision, resources.

## Funding

This work was supported by the Federal Ministry of Education and Research of the Grant Project BEPROF@BHV—Hochschule Bremerhaven. The funders had no role in the design and analysis of the research or the decision to publish the work.

## Conflicts of Interest

The authors declare no conflicts of interest.

## Supporting information




**Supplementary Material**: crf370495‐sup‐0001‐SuppMat.docx

## Data Availability

The tables and figures in the Supporting Information contain the method and databases supporting this article.
