# Supplementary material for: Recent Advances in Egg White Biotechnology, Microbiology, and Quality
Source: Compr Rev Food Sci Food Saf. 2026 May 18;25:e70495. doi: 10.1111/1541-4337.70495 (PMC13184442; doi:10.1111/1541-4337.70495)
Supplement: Supplementary file 1 — Supplementary Material: crf370495‐sup‐0001‐SuppMat.docx [file CRF3-25-e70495-s001.docx]

**Supplementary material**

**Recent advances in egg white biotechnology, microbiology, and quality**

**Supplement 1- Protocol**

**S1.1 Background and objective**

Egg white, also known as albumen, is a highly functional component of hen's eggs with wide applications in the food industry, pharmaceuticals, and biotechnology. Due to its high protein content, especially ovalbumin, lysozyme, and ovotransferrin, egg white offers excellent techno-functional properties such as foaming, emulsification, and gel formation. At the same time, it has a certain degree of natural preservation owing to antimicrobial proteins. The microbiological safety and quality of egg white are closely linked to its processing, storage, and use in various applications. While pasteurized liquid egg white is an established product, alternative processes create new challenges and opportunities regarding microbial contamination, especially by heat-resistant or contaminating microorganisms. Simultaneously, the use of selected microorganisms—such as lactic acid bacteria—opens up innovative approaches for natural preservation or functionalization. This review aims to summarize the current state of the literature, and systematically present which preservation methods are used, how they can be classified, and for which quality parameters they are suitable.

**S1.2 Methodology**

This review offers a comprehensive summary of the latest knowledge on microbiology, quality attributes, and biotechnological applications of egg white. It follows the PRISMA-ScR (Preferred Reporting Items for Systematic Reviews and Meta-Analyses extension for Scoping Reviews) guidelines. The protocol is based on the Tricco et al. (2018) checklist (see Appendix 2 - PRISMA-ScR Checklist).

**S1.2.1 Databases and search strategy**

Relevant scientific literature was identified through an extensive search of the following databases: PubMed, Web of Science, and Espacenet. Searches were conducted between January 2023 and May 2025. Other sources included technical reports, regulatory documents, and book chapters (SUUB or from authorities) where relevant. The search strategy was adapted to the respective database as required (see A3). In response to reviewer comments, additional relevant studies were identified and assessed for eligibility in February 2026 using the predefined inclusion and exclusion criteria. These studies met all inclusion criteria and were therefore incorporated into the final synthesis. This process is transparently documented in the PRISMA flow diagram.

The following search terms and combinations were used: “antimicrobial defenses egg“ OR „egg white“ OR „bioactive peptides from egg“ OR „new processing technologies egg“ OR „enhanced foaming egg“ OR „functional proteins egg white“ OR „processing technologies egg“ OR „processing technologies egg white“ OR „spray drying egg white“ OR „egg white fermentation“ OR „ultrasound egg white“ OR „Cold Plasma egg white“ OR „microorganism egg white“ OR „egg white high pressure processing“ OR „microorganism egg“ OR „proteins egg white“ OR „fungi egg white“ OR „molds egg“ OR „virus egg“ OR „egg white novel processing methods“ OR „egg white processing“.

**S1.2.2 Inclusion and exclusion criteria**

The search was limited to publications in English or German. Preference was given to work from the last 10 years, but older sources were included if they were of fundamental importance. To ensure comprehensive coverage, the reference lists of key papers were manually reviewed, and additional relevant publications were added when appropriate. This review only covered articles on hens' eggs. Articles primarily related to medical applications, products other than food (e.g., films), or genetic modifications were excluded unless directly related to the topic.

**S1.3 Assessment of risk of bias**

The risk of bias was not assessed because this is typically only used for medical studies.

**S1.3.4 Selection and evaluation of literature**

The literature was selected in two stages. First, titles and abstracts were screened to evaluate relevance and appropriateness. In the second stage, full texts were examined. The final selection was based on relevance, scientific quality, and originality. Special attention was given to studies with microbiological, biotechnology, or technological importance. The results of the literature search were organized thematically and contextualized. Additionally, relevant sources from the references of selected literature and recommendations were included.

**S1.4 Results**

A comprehensive literature search was conducted up to May 2025, resulting in 31934 records, of which were unique after removing duplicates. From these unique records, 31500 were excluded during the title and abstract screening, leaving 424 records for full-text review. Of these, 123 met the inclusion criteria for the review (Fig. 1-A4). Additional records were identified by examining references of eligible papers, through recommendations, or via the catalog of the Bremen State and University Library. The final number of documents included in this review was 330, published between 2012 and 2025.

**Supplement 2 - PRISMA-ScR Checklist**

Table 1-S2: Preferred Reporting Items for Systematic Reviews and Meta-Analyses extension for Scoping Reviews (PRISMA-ScR) Checklist (N/A = Not Applied)

| **SECTION** | **ITEM** | **PRISMA-ScR CHECKLIST ITEM** | **REPORTED ON PAGE #** |
| --- | --- | --- | --- |
| **TITLE** | | | |
| Title | 1 | Identify the report as a scoping review. | Page 1 |
| **ABSTRACT** | | | |
| Structured summary | 2 | Provide a structured summary that includes (as applicable): background, objectives, eligibility criteria, sources of evidence, charting methods, results, and conclusions that relate to the review questions and objectives. | Page 1 |
| **INTRODUCTION** | | | |
| Rationale | 3 | Describe the rationale for the review in the context of what is already known. Explain why the review questions/objectives lend themselves to a scoping review approach. | Page 2-4 and Appendix A1 |
| Objectives | 4 | Provide an explicit statement of the questions and objectives being addressed concerning their key elements (e.g., population or participants, concepts, and context) or other relevant key elements used to conceptualize the review questions and/or objectives. | Page 2-4 and Appendix A1 |
| **METHODS** | | | |
| Protocol and registration | 5 | Indicate whether a review protocol exists; state if and where it can be accessed (e.g., a Web address); and if available, provide registration information, including the registration number. | see protocol in Appendix A1; the protocol has not been registered |
| Eligibility criteria | 6 | Specify characteristics of the sources of evidence used as eligibility criteria (e.g., years considered, language, and publication status), and provide a rationale. | see protocol in Appendix A1 |
| Information sources* | 7 | Describe all information sources in the search (e.g., databases with dates of coverage and contact with authors to identify additional sources), as well as the date the most recent search was executed. | see protocol in Appendix A1 & A3 |
| Search | 8 | Present the full electronic search strategy for at least 1 database, including any limits used, such that it could be repeated. | see protocol in ppendix A3 |
| Selection of sources of evidence† | 9 | State the process for selecting sources of evidence (i.e., screening and eligibility) included in the scoping review. | see protocol in Appendix A1 |
| Data charting process‡ | 10 | Describe the methods of charting data from the included sources of evidence (e.g., calibrated forms or forms that have been tested by the team before their use, and whether data charting was done independently or in duplicate) and any processes for obtaining and confirming data from investigators. | N/A |
| Data items | 11 | List and define all variables for which data were sought and any assumptions and simplifications made. | N/A |
| Critical appraisal of individual sources of evidence§ | 12 | If done, provide a rationale for conducting a critical appraisal of included sources of evidence; describe the methods used and how this information was used in any data synthesis (if appropriate). | N/A |
| Synthesis of results | 13 | Describe the methods of handling and summarizing the data that were charted. | Appendix A1 |
| **RESULTS** | | | |
| Selection of sources of evidence | 14 | Give numbers of sources of evidence screened, assessed for eligibility, and included in the review, with reasons for exclusions at each stage, ideally using a flow diagram. | See Appendix A4 |
| Characteristics of sources of evidence | 15 | For each source of evidence, present characteristics for which data were charted and provide the citations. | N/A |
| Critical appraisal of sources of evidence | 16 | If done, present data on critical appraisal of included sources of evidence (see item 12). | N/A |
| Results of individual sources of evidence | 17 | For each included source of evidence, present the relevant data that were charted that relate to the review questions and objectives. | N/A |
| Synthesis of results | 18 | Summarize and/or present the charting results as they relate to the review questions and objectives. | N/A |
| **DISCUSSION** | | | |
| Summary of evidence | 19 | Summarize the main results (including an overview of concepts, themes, and types of evidence available), link to the review questions and objectives, and consider the relevance to key groups. | N/A |
| Limitations | 20 | Discuss the limitations of the scoping review process. | N/A |
| Conclusions | 21 | Provide a general interpretation of the results concerning the review questions and objectives, as well as potential implications and/or next steps. | N/A |
| **FUNDING** | | | |
| Funding | 22 | Describe sources of funding for the included sources of evidence, as well as sources of funding for the scoping review. Describe the role of the funders of the scoping review. | Page |

JBI = Joanna Briggs Institute; PRISMA-ScR = Preferred Reporting Items for Systematic reviews and Meta-Analyses extension for Scoping Reviews.

* Where *sources of evidence* (see second footnote) are compiled from, such as bibliographic databases, social media platforms, and websites.

† A more inclusive/heterogeneous term used to account for the different types of evidence or data sources (e.g., quantitative and/or qualitative research, expert opinion, and policy documents) that may be eligible in a scoping review as opposed to only studies. This is not to be confused with *information sources* (see first footnote).

‡ The frameworks by Arksey and O’Malley (6) and Levac and colleagues (7) and the JBI guidance (4, 5) refer to the process of data extraction in a scoping review as data charting*.*

§ The process of systematically examining research evidence to assess its validity, results, and relevance before using it to inform a decision. This term is used for items 12 and 19 instead of "risk of bias" (which is more applicable to systematic reviews of interventions) to include and acknowledge the various sources of evidence that may be used in a scoping review (e.g., quantitative and/or qualitative research, expert opinion, and policy documents).

**Supplement 3 - Search strings per database (documented in detail)**

**S3.1 Pubmed**

Search string: (All fields) antimicrobial defenses egg OR egg white OR bioactive peptides from egg OR new processing technologies egg OR enhanced foaming egg OR functional proteins egg white OR processing technologies egg OR spray drying egg white OR egg white fermentation OR ultrasound egg white OR Cold Plasma egg white OR microorganism egg white OR egg white high-pressure processing OR microorganism egg OR proteins egg white OR fungi egg white OR molds egg OR virus egg OR egg white novel processing methods OR egg white processing

Filter: Language: English or German

- Date filter: 2014-2025
- Document type: Article, Review, and Conference Paper

Date of the last search per database: 30.05.2025

Export format: PubMed

Software for literature management: Literature was selected and organized with the help of the reference management software Citavi.

**S3.2 Web of Science**

Search string (All fields):

(All fields) antimicrobial defenses egg, OR egg white OR bioactive peptides from egg OR new processing technologies egg OR enhanced foaming egg OR functional proteins egg white OR processing technologies egg

(All fields) processing technologies egg white OR spray drying egg white OR egg white fermentation OR ultrasound egg white OR Cold Plasma egg white OR microorganism egg white OR egg white high pressure processing OR microorganism egg OR proteins egg white OR fungi egg white OR molds egg OR virus egg OR egg white novel processing methods OR egg white processing

Filter:

- Language: English or German
- Date filter: 2014-2025
- Document type: Article, Review, and Conference Paper

Date of the last search per database: 31.05.2025

Export format: BibTex

Software for literature management: Literature was selected and organized with the help of the reference management software Citavi.

**S3.4 Espacenet**

Search string (All fields): fermented egg white

Filter:

- Language: English or German
- Date filter: 2015-2025
- Document type: PDF

Date of the last search per database: 31.05.2025

Export format: csv

Software for literature management: Literature was selected and organized with the help of the reference management software Citavi.

**S3.5 Staats- und Universitätsbibliothek Bremen (SUUB)**

Search string: (All Fields)

Filter:

- Language: English or German
- Date filter: 2015-2025
- Format: Online

Date of the last search per database: 30.12.2024

Export format: PDF

Software for literature management: Literature was selected and organized with the help of the reference management software Citavi.

**Supplement 4 – PRISMA Flow Diagram**


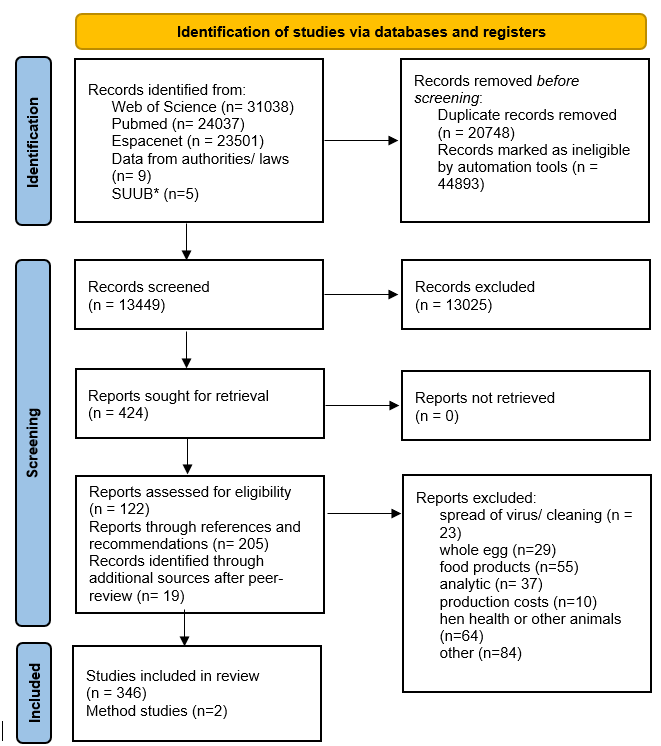


Figure 1- A4 PRISMA 2020 flow diagram for new systematic reviews, which included searches of databases and registers (modified from Page et al. 2021)
